# Novel Investigation of Higher Order Spectral Technologies for Fault Diagnosis of Motor-Based Rotating Machinery

**DOI:** 10.3390/s23073731

**Published:** 2023-04-04

**Authors:** Tomasz Ciszewski, Len Gelman, Andrew Ball, Abdulmumeen Onimisi Abdullahi, Biebele Jamabo, Michal Ziolko

**Affiliations:** 1 Faculty of Electrical Engineering, Gdynia Maritime University, 81-255 Gdynia, Poland; 2 School of Computing and Engineering, University of Huddersfield, Huddersfield HD1 3DH, UK; 3 Faculty of Electrical and Control Engineering, Gdańsk University of Technology, 80-233 Gdansk, Poland

**Keywords:** signal processing, damage diagnosis, motor current signature analysis, induction motor, bearings

## Abstract

In the last decade, research centered around the fault diagnosis of rotating machinery using non-contact techniques has been significantly on the rise. For the first time worldwide, innovative techniques for the diagnosis of rotating machinery, based on electrical motors, including generic, nonlinear, higher-order cross-correlations of spectral moduli of the third and fourth order (CCSM3 and CCSM4, respectively), have been comprehensively validated by modeling and experiments. The existing higher-order cross-correlations of complex spectra are not sufficiently effective for the fault diagnosis of rotating machinery. The novel technology CCSM3 was comprehensively experimentally validated for induction motor bearing diagnosis via motor current signals. Experimental results, provided by the validated technology, confirmed high overall probabilities of correct diagnosis for bearings at early stages of damage development. The novel diagnosis technologies were compared with existing diagnosis technologies, based on triple and fourth cross-correlations of the complex spectra. The comprehensive validation and comparison of the novel cross-correlation technologies confirmed an important non-traditional novel outcome: the technologies based on cross-correlations of spectral moduli were more effective for damage diagnosis than the technologies based on cross-correlations of the complex spectra. Experimental and simulation validations confirmed a high probability of correct diagnosis via the CCSM at the early stage of fault development. The average total probability of incorrect diagnosis for the CCSM3 for all experimental results of 8 tested bearings, estimated via 6528 diagnostic features, was 1.475%. The effectiveness gains in the total probability of incorrect diagnosis for the CCSM3 in comparison with the CCCS3 were 26.8 for the experimental validation and 18.9 for the simulation validation. The effectiveness gains in the Fisher criterion for the CCSM3 in comparison with the CCCS3 were 50.7 for the simulation validation and 104.7 for the experimental validation.

## 1. Introduction

Pumps, gear motors, and cranes are examples of IM applications. Most IMs are required to operate for a long time between maintenance actions. That is why effective condition monitoring is needed in industrial applications of motors [1,2,3,4,5,6,7,8,9], gear motors [10,11,12,13,14,15,16,17], and other structures. Proper fault diagnosis and fault isolation of key components is crucial for continuity of IM operations. Bearings are among the most common parts susceptible to damage in an IM, accounting for 42% of all failures [1]. That is why their efficient diagnosis is highly desired. This need is the reason for constant development of new and more effective diagnostic and condition monitoring techniques. Recent work on this topic more and more frequently highlights higher order spectral signal processing techniques as being very efficient, e.g., ref. [1,2,3,4,5]. Diagnostic methods based on IM stator current are becoming more popular every year [6,7,8,9]. Gangsar et al. [8] presented an extended and complete state-of-the-art review of various IM faults, detection, identification, and diagnosis.

For bearing diagnosis in an industrial environment, vibration-based methods are still the most used. However, they require the installation of expensive vibration sensors on the engine, making these methods invasive, inconvenient, and costly. vibration methods are well known and have been used for a long time to detect bearing damage [18,19,20,21,22,23,24,25,26]. vibration methods are still researched to achieve better damage diagnosis, mainly in terms of signal processing technique improvements.

Motor current signature analysis (MCSA) is the subject of much up-to-date research, as it has become a non-invasive substitute to vibration-based methods for IM bearing diagnosis. This method significantly reduces the cost of diagnosis, as it does not require the installation of expensive piezoelectric accelerometers on a motor. MCSA can be performed remotely, as the IM current can be measured with only access to the power supply line of a motor. IM current signals are frequently used for detecting electrical faults in motors and faults in coupled gearboxes [27]. Early diagnosis of IM bearing faults via MCSA is a difficult task due to the low signal-to-noise ratio (SNR) of IM current spectral components, which contain the information on the bearing conditions [6,7,8,28,29,30,31,32,33,34,35]. The instantaneous changes in the shape of the IM current due to bearing damage are practically undetectable; therefore, most methods based on MCSA use current spectral visualization. The low SNR of bearing diagnostic components in the IM current spectrum may cause a lot of spectral components of various origins to occur in the same frequency areas. Therefore, it is crucial to estimate instantaneous motor speed and the frequencies related to bearing damage with a high precision in order to track damage-related components in the time—frequency domain. Rotor speed information can be acquired from the IM current spectrum and rotor slot harmonics (RSH) with high accuracy. The method of IM rotor speed estimation based on RSH frequency demodulation is introduced in ref. [35]; this method gives precise results for steady-state and non-stationary conditions of IM operations.

Spectral component frequencies used in IM bearing diagnosis are estimated using models that have been improved over time. Studies [28,36] have revealed that the previous MCSA solutions were based on an incomplete model for characteristic frequency estimations. The authors of ref. [36] considered the changes in torque caused by bearing damage. It has been shown that IM torque oscillations have a significant impact on the characteristic frequencies. In cases of inner race or rolling element damage, new models for characteristic frequency estimation are different than earlier versions used in research by ref. [36]. The authors of ref. [28] present a dynamic model of a rotor with damaged bearings in order to numerically simulate the air gap oscillations. The IM current modeling was based on the magnetic circuit model.

The other approach to monitoring IM bearing condition is presented in ref. [6,31], in which the authors propose to use vibration-based methods for bearing characteristic frequency estimation. The envelope of the vibration signal is used for characteristic component detection. Detected components are then tracked in the IM current spectrum and changes in their amplitude are used for bearing diagnosis.

The authors of ref. [32] proposed to use a diagnostic indicator based on a new fault-excited harmonic distortion. The IM model developed in ref. [32] showed a clear relationship between the value of proposed diagnostic indicator and bearing fault severity. However, this method was tested only for one outer race defect for a specific type of IM. Other types of IM will require the creation of other models. The model used in ref. [32] did not consider saturation of the IM core and considered only radial eccentricities.

To improve the reliability of MCSA-based IM bearing damage diagnosis, new and robust signal processing techniques are sought. Recent research in this area considered using the wavelet transform [7,22,23,24,25,36,37,38], higher-order spectral techniques [1,2,3,4,5,20,33,34], and spectral kurtosis [19,21,39].

In ref. [7], the continuous wavelet transform was used with MCSA to detect damage in the bearing installed not in motor itself but in the IM load. In this case, the damage did not influence the IM air gap, only IM torque oscillations related to the damage affected the IM current. In the presented case, the characteristic spectral components of the IM current were barely detectable with IM current spectrum-based methods [7].

MCSA-based higher-order spectral techniques were employed in ref. [1,2,4,33,34] for IM damage detection. These methods were able allow to detect the interactions between the spectral components. It has been shown that higher-order spectral techniques enable not only fault detection at an early stage of development but also an indication of fault severity.

An MCSA-based method that involved instantaneous frequency analysis was proposed in ref. [40]. In ref. [30], an MCSA-based method was proposed for IM bearing diagnosis. The method was tested on a small number of bearing outer race faults in light load conditions of tested IMs. Classical MCSA-based methods mostly fail in detecting this type of damage due to poor SNR [30].

The Park vector approach used in MCSA-based IM bearing diagnosis allows the enhancement of components related to bearing damage, which slightly increases the poor SNR. After Park vector estimation, various sophisticated methods of signal processing can be used for the detection of fault-related components [30,35]. This approach can be applied for the detection of bearing faults [30,31,35,41,42,43,44,45] as well as other IM faults, such as broken rotor bars [46,47,48,49,50,51,52] and stator winding faults [53,54,55,56].

The authors of ref. [1,2] proposed novel higher-order spectral signal processing techniques applied in IM current signal processing for the purpose of IM bearing diagnosis. However, the validation of these techniques is not sufficient in both aforementioned studies. Ref. [1] did not include validation via simulation; the technique proposed in ref. [1] was validated only by experimental trials. In ref [2], validation is made via simulation; however, the experimental validation was based only on one diagnosis case. The purpose of this paper was to essentially decrease the validation insufficiency via comprehensive validation and compare the two higher-order spectral techniques presented in ref. [1,2]. The validation and comparison were performed via comprehensive modeling and experimental trials.

Thus, the main novelties here include novel validation by comprehensive modeling and experiments of novel, nonlinear, higher-order cross-correlations for damage diagnosis of rotating machinery based on electrical motors.

Other novelties of the research are as follows:

- novel comparison of the CCSM3 and CCCS3 technologies via comprehensive modeling trials
- novel comparison of the CCSM4 and CCCS4 technologies via comprehensive modeling trials
- novel comparison of the CCSM3 technologies and CCCS3 technologies via comprehensive experimental trials
- The objectives of the research are as follows:
- perform a novel comprehensive validation of the CCSM3 and CCSM4 via modeling trials for pristine and faulty mechanical components
- perform a novel comparison of the CCSM3 with the CCCS3 and the CCSM4 with the CCCS4 via modeling trials for pristine and faulty mechanical components
- perform novel comprehensive validation of the CCSM3 via experimental trials for pristine and faulty motor bearings, using motor current signal processing
- perform a novel comparison of the CCSM3 with the CCCS3 via experimental trials for pristine and faulty motor bearings, using motor current signal processing

## 2. Higher-Order Spectral Cross-Correlations

The first higher-order spectral signal processing technologies examined in this paper are the cross-correlations of spectral moduli (CCSM) proposed in ref. [1]. The CCSM of order *n* is described by Equation (1) [1].

(1) $$CCSM(f_{1},f_{2},\ldots ,f_{n},t)=\frac{\sum_{j=1}^{I}\left[\left({mZ}_{f_{1}}^{j}(t)\right)\left({mZ}_{f_{2}}^{j}(t)\right)\ldots ({mZ}_{f_{n}}^{j}(t))\right]}{\sqrt[{n}]{\sum_{j=1}^{I}\left[{\left|{mZ}_{f_{1}}^{j}(t)-\bar{{mZ}_{f_{1}}(t)}\right|}^{n}\right]}\sqrt[{n}]{\sum_{j=1}^{I}\left[{\left|{mZ}_{f_{2}}^{j}(t)-\bar{{mZ}_{f_{2}}(t)}\right|}^{n}\right]}\ldots \sqrt[{n}]{\sum_{j=1}^{I}\left[{\left|{mZ}_{f_{n}}^{j}(t)-\bar{{mZ}_{f_{n}}(t)}\right|}^{n}\right]}}$$

where I is the total segment number and ${mZ}_{f_{n}}^{j}$ is the spectral modulus, related to any complex time–frequency transform or any complex frequency transform of time segment j for frequency $f_{n}$.

The CCSM of orders three (CCSM3) and four (CCSM4) are expressed by Equations (2) and (3), respectively [1]:

(2) $$CCSM3(f_{1},f_{2},f_{3},t)=\frac{\sum_{j=1}^{I}\left[\left({mZ}_{f_{1}}^{j}(t))({mZ}_{f_{2}}^{j}(t))({mZ}_{f_{3}}^{j}(t)\right)\right]}{\sqrt[{3}]{\sum_{j=1}^{I}\left[{\left|{mZ}_{f_{1}}^{j}(t)-\bar{{mZ}_{f_{1}}(t)}\right|}^{3}\right]}\sqrt[{3}]{\sum_{j=1}^{I}\left[{\left|{mZ}_{f_{2}}^{j}(t)-\bar{{mZ}_{f_{2}}(t)}\right|}^{3}\right]}\sqrt[{3}]{\sum_{j=1}^{I}\left[{\left|{mZ}_{f_{3}}^{j}(t)-\bar{{mZ}_{f_{3}}(t)}\right|}^{3}\right]}}$$

(3) $$\begin{array}{c} CCSM4(f_{1},f_{2},f_{3},f_{4},t)=\\ \frac{\sum_{j=1}^{I}\left[\left({mZ}_{f_{1}}^{j}(t))({mZ}_{f_{2}}^{j}(t))({mZ}_{f_{3}}^{j}(t))({mZ}_{f_{4}}^{j}(t)\right)\right]}{\sqrt[{4}]{\sum_{j=1}^{I}\left[{\left|{mZ}_{f_{1}}^{j}(t)-\bar{{mZ}_{f_{1}}(t)}\right|}^{4}\right]}\sqrt[{4}]{\sum_{j=1}^{I}\left[{\left|{mZ}_{f_{2}}^{j}(t)-\bar{{mZ}_{f_{2}}(t)}\right|}^{4}\right]}\sqrt[{4}]{\sum_{j=1}^{I}\left[{\left|{mZ}_{f_{3}}^{j}(t)-\bar{{mZ}_{f_{3}}(t)}\right|}^{4}\right]}\sqrt[{4}]{\sum_{j=1}^{I}\left[{\left|{mZ}_{f_{4}}^{j}(t)-\bar{{mZ}_{f_{4}}(t)}\right|}^{4}\right]}} \end{array}$$

The second higher-order spectral signal processing technologies examined in this paper are the cross-correlation of complex spectra (CCCS). An unnormalized version of these technologies is proposed in ref. [2]. Normally, the normalization of higher-order correlations of random variables is based on the absolute central moments of order n, proposed and widely investigated in many mathematical works, e.g., ref. [57,58,59,60,61]. The CCCS of order *n* is described by Equation (4):

(4) $$CCCS(f_{1},f_{2},\ldots ,f_{n},t)=\frac{\sum_{j=1}^{I}\left[\left({cZ}_{f_{1}}^{j}(t)\right)\left({cZ}_{f_{2}}^{j}(t)\right)\ldots conj(cZ_{f_{n}}^{j}(t))\right]}{\sqrt[{n}]{\sum_{j=1}^{I}\left[{\left|{cZ}_{f_{1}}^{j}(t)-\bar{{cZ}_{f_{1}}(t)}\right|}^{n}\right]}\sqrt[{n}]{\sum_{j=1}^{I}\left[{\left|{cZ}_{f_{2}}^{j}(t)-\bar{{cZ}_{f_{2}}(t)}\right|}^{n}\right]}\ldots \sqrt[{n}]{\sum_{j=1}^{I}\left[{\left|{cZ}_{f_{n}}^{j}(t)-\bar{{cZ}_{f_{n}}(t)}\right|}^{n}\right]}}$$

where ${cZ}_{f_{n}}^{j}$ is the complex spectral component, related to any complex time–frequency transform or any complex frequency transform of time segment j for frequency $f_{n}$.

The CCCS of orders three (CCCS3) and four (CCCS4) are expressed by Equations (5) and (6), respectively.

(5) $$CCCS3(f_{1},f_{2},f_{3},t)=\frac{\sum_{j=1}^{I}\left[\left({cZ}_{f_{1}}^{j}(t))({cZ}_{f_{2}}^{j}(t))conj({cZ}_{f_{3}}^{j}(t)\right)\right]}{\sqrt[{3}]{\sum_{j=1}^{I}\left[{\left|{cZ}_{f_{1}}^{j}(t)-\bar{{cZ}_{f_{1}}(t)}\right|}^{3}\right]}\sqrt[{3}]{\sum_{j=1}^{I}\left[{\left|{cZ}_{f_{2}}^{j}(t)-\bar{{cZ}_{f_{2}}(t)}\right|}^{3}\right]}\sqrt[{3}]{\sum_{j=1}^{I}\left[{\left|{cZ}_{f_{3}}^{j}(t)-\bar{{cZ}_{f_{3}}(t)}\right|}^{3}\right]}}$$

(6) $$\begin{array}{c} CCCS4(f_{1},f_{2},f_{3},f_{4},t)=\\ \frac{\sum_{j=1}^{I}\left[\left({cZ}_{f_{1}}^{j}(t))({cZ}_{f_{2}}^{j}(t))({cZ}_{f_{3}}^{j}(t))conj({cZ}_{f_{4}}^{j}(t)\right)\right]}{\sqrt[{4}]{\sum_{j=1}^{I}\left[{\left|{cZ}_{f_{1}}^{j}(t)-\bar{{cZ}_{f_{1}}(t)}\right|}^{4}\right]}\sqrt[{4}]{\sum_{j=1}^{I}\left[{\left|{cZ}_{f_{2}}^{j}(t)-\bar{{cZ}_{f_{2}}(t)}\right|}^{4}\right]}\sqrt[{4}]{\sum_{j=1}^{I}\left[{\left|{cZ}_{f_{3}}^{j}(t)-\bar{{cZ}_{f_{3}}(t)}\right|}^{4}\right]}\sqrt[{4}]{\sum_{j=1}^{I}\left[{\left|{cZ}_{f_{4}}^{j}(t)-\bar{{cZ}_{f_{4}}(t)}\right|}^{4}\right]}} \end{array}$$

In order to estimate diagnostic features, the CCSM3/CCSM4 and CCCS3/CCCS4, the discrete time domain signal has to be divided into time segments of a predefined duration. The following step is to perform the selected frequency transform or time–frequency transform of each time segment. In this paper, the Fourier transform is used. The final step is to estimate the CCSM3/CCSM4 and CCCS3/CCCS4 via Equations (2), (3), (5) and (6), respectively.

To evaluate the diagnostic effectiveness of the technologies, histograms of the considered diagnostic features under various settings are evaluated. Then, each histogram is approximated by the normal (i.e., Gaussian) probability density function. The normal probability density function of random variable X with mean (μ) and standard deviation (σ) is stated as follows:

(7) $$PDF\left(X\right)=\frac{1}{\sigma \sqrt{2\pi }}e^{\frac{-{\left(x-\mu \right)}^{2}}{2\sigma^{2}}}$$

To compare the CCSM with the CCCS, two estimates related to a diagnosis accuracy are used. The first estimate is the total probability of correct diagnosis (TPCD) described by Equation (8) and the second estimate is the Fisher criterion (FC) described by Equation (9). A threshold-based decision-making rule (an aqua line is used to indicate the diagnostic threshold) is employed via the Bayesian criterion [62] for the decision making for each one-dimensional diagnostic feature.

(8) $$TPCD=\frac{rN+prN}{rNt+prNt}100\%$$

where *rN* and *prN* are the total numbers of correct diagnosis for the defective bearing and new bearing, respectively; *rNt* and *prNt* are the total numbers of diagnostic features for the defective bearing and new bearing, respectively.

(9) $$FC=\frac{{\left|{bm}_{1}-bm_{2}\right|}^{2}}{\sigma_{1}^{2}+\sigma_{2}^{2}}$$

where ${bm}_{1}$ is the mean value of diagnostic features for the new bearing, ${bm}_{2}$ is the mean value of diagnostic features for the defective bearing, $\sigma_{1}^{2}$ is the variance of diagnostic features for the new bearing, and $\sigma_{2}^{2}$ is the variance of diagnostic features for the defective bearing.

## 3. Technology Validation via Modeling

To compare the proposed CCSM and CCCS techniques, healthy and damaged mechanical components were modeled. A generic bilinear oscillator was used to model these components. A generic dynamic bi-linear oscillator used for modeling is widely employed for modeling various electromechanical components of industrial electromechanical systems and complex structures, e.g., sub-harmonic resonances of offshore structures [63], loading towers [64], components with clearances [65], and the following components with local damage: rolling element bearings with local damage in outer races, inner races, cages, and rolling elements [66,67], gearboxes with local damage in gear teeth [68], and rotating blades and shafts of turbomachinery with fatigue damage [69,70,71,72,73,74,75]. The model describes “in-process damage” as local damage such as fatigue cracks, pitting, local scratches, etc. Thus, this model is suitable for the investigation proposed in the paper.

The equation for the bilinear oscillator is shown below [73]:

(10) $$\frac{d^{2}y}{dt^{2}}+2h\frac{dy}{dt}+{\omega^{2}}_{s}x\left(t\right)=Acos\left(\omega t+\varphi \right),\;x\geq 0$$

(11) $$\frac{d^{2}y}{dt^{2}}+2h\frac{dy}{dt}+{\omega^{2}}_{c}x\left(t\right)=Acos\left(\omega t+\varphi \right),\;x<0$$

(12) $$x\left(t\right)=\frac{X\left(t\right)}{m},\;h=\frac{c}{2m},\;\omega_{s}=\sqrt{\frac{k_{s}}{m}},\;\omega_{c}=\sqrt{\frac{k_{c}}{m}},\;A=\frac{A_{1}}{m}$$

where X(t) is the displacement, x(t) is the normalized displacement, m is the mass, h is damping, c is the damping coefficient, $k_{s}$ is the stiffness of positive displacement, $k_{c}$ is the stiffness of negative displacement, $A_{1}$ is the constant amplitude of an input sinusoidal signal (i.e., the excitation), A is the normalized amplitude of the excitation, ω is the constant angular frequency of the excitation, and φ is the random initial phase of the excitation. $\omega_{s}$ and $\omega_{c}$ are the resonance frequencies at positive and negative displacements.

The random initial phase is uniformly distributed in the range [0; 2π]. Equations (10)–(12) are used to describe a mechanical component with and without damage. The stiffness of positive displacement $k_{s}$ and the stiffness of negative displacement $k_{c}$ are equal for an undamaged component, whereas for a component with damage, the stiffness of positive displacement $k_{s}$ and the stiffness of negative displacement $k_{c}$ are different. A relative damage severity is characterized by the stiffness ratio $k^{*}=\frac{k_{c}-k_{s}}{k_{c}}$. The resonance frequency of a component with damage is calculated via Equation (13) below [73]:

(13) $$\omega_{o}=\omega_{c}\frac{2\times \sqrt{1-k^{*}}}{1+\sqrt{1-k^{*}}}$$

A total of 300 time domain realizations were simulated via Equations (10)–(13) to investigate the cross-correlation techniques, including:

- for healthy components
- for damaged components with relative damage severity of 5%
- for damaged components with relative damage severity of 10%

These signals were simulated using resonance frequencies $\omega_{c}$ of 14 and 30 Hz one set being undamaged and another set having damage levels of 5% and 10%. The signals with a resonance frequency $\omega_{c}$ of 14 Hz were sampled by a sampling frequency of 340 Hz, while those with a resonance frequency $\omega_{c}$ of 30 Hz were sampled with a sampling frequency of 720 Hz. All signals for healthy and damaged components were simulated with a damping of 2.45 and the time duration of each simulated signal was 80 s.

The following parameters were used to evaluate the cross-correlation techniques: the internal window was 2 s, the frequency resolution was 0.5 Hz, the time window was the Hanning window, and the overlapping of internal windows was 0%. Gaussian white noise was added to each simulated signal to simulate an early stage of damage.

**Figure 1:**
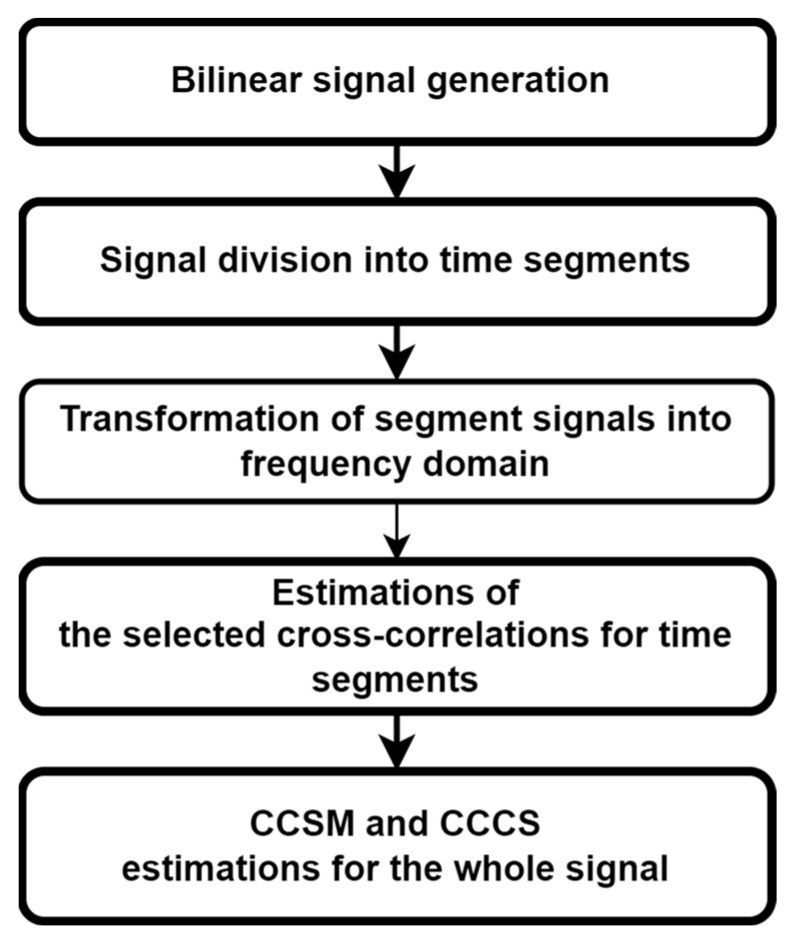
The modeling flow chart for the CCSM and CCCS of the third and fourth orders.

The modeling flow chart for the CCSM3/CCCS3 and CCSM4/CCCS4 is given below in Figure 1.

### 3.1. Validation of Third Order Cross-Correlations via Modeling

The CCSM3 and CCCS3 are functions of three independent frequencies. To estimate the diagnostic effectiveness of the CCSM3 and CCCS3, four sets of harmonics were investigated using the simulated signals from components with resonance frequencies $\omega_{c}$ of 14 and 30 Hz. The investigated sets of harmonics of component resonance oscillations were as follows: (1,1,3), (1,3,5), (1,2,4), and (1,2,5). The triple cross-correlations for harmonic set (1,1,3) were estimated for the three resonance harmonics: the fundamental resonance harmonic that was employed twice and the third resonance harmonic. The triple cross-correlations for harmonic set (1,3,5) were estimated for the three resonance harmonics: the fundamental resonance harmonic, the third resonance harmonic, and the fifth resonance harmonic. The triple cross-correlations for harmonic set (1,2,4) were estimated for the three resonance harmonics: the fundamental resonance harmonic, the second resonance harmonic, and the fourth resonance harmonic of the component resonance oscillations. Finally, the triple cross-correlations for harmonic set (1,2,5) were estimated for the three resonance harmonics: the fundamental resonance harmonic, the second resonance harmonic, and the fifth resonance harmonic.

**Figure 2:**
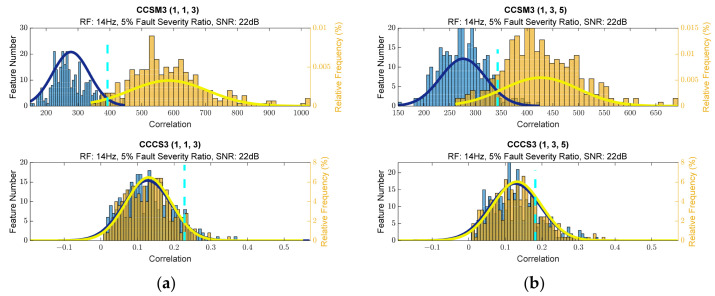
(**a**) Histograms of diagnostic features for (1,1,3) harmonic set. (**b**) Histogram of diagnostic features for (1,3,5) harmonic set.

To estimate the CCSM3 and CCCS3, SNR of 22 dB was used for 5% relative damage severity and SNR of 18 dB was used for 10% relative damage severity. Figure 2a,b show histograms for the selected diagnostic features. The histograms for additional frequency sets, taken into consideration for the CCSM3 and CCCS3, are presented in Figure A36, Figure A37, Figure A38 and Figure A39 in Appendix A. The superiority of the CCSM3 over the CCCS3 was also observed on these histograms.

The main parameter that affects an accurate histogram evaluation is the bin number. The bin number is selected to accurately extract the shape of the histograms, using the following methodology. For each pair of the obtained modeling histograms in Section 3.1 and 3.2, the total number of diagnostic features for the damaged and undamaged conditions were both 300. The standard “Rule of Thumb” is to divide a feature range into k equal bins, where k is the square root of a feature number. So, for each histogram (i.e., for the damaged and undamaged conditions), 18 bins were needed. Taking into account a distance between histograms for the undamaged and damaged conditions, a “safety factor” of 1.25 was applied and the obtained total number of bins was 45. The bin range for each pair of histograms was defined based on a minimum diagnostic feature value for the undamaged conditions and a maximum diagnostic feature value for the damaged conditions. Once the bin range was defined, the bin size was automatically defined based on the defined bin range and selected number of bins. If we choose a bin number that is too big for a given feature number, the bar height at each bin will suffer significant statistical fluctuation due to the paucity of samples in each bin. If we choose a bin number that is too small, the histogram cannot represent the shape of the underlying distribution because the resolution is insufficient.

**Figure 3:**
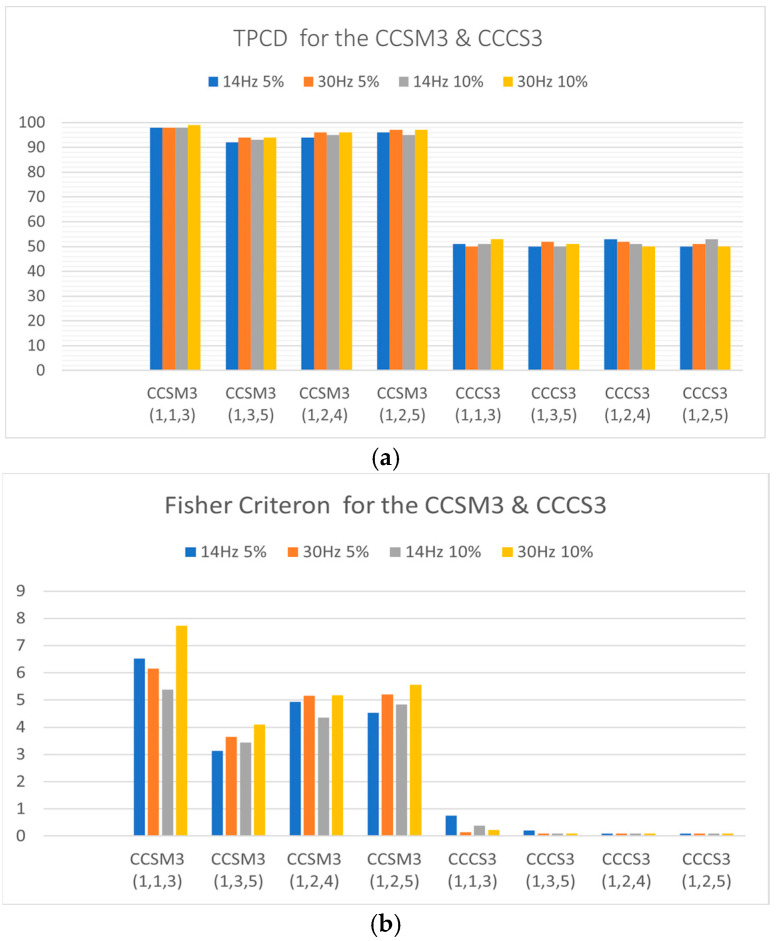
(**a**) The TPCDs for all investigated cases for the CCSM3 and CCCS3. (**b**) The Fisher criteria for all investigated cases for the CCSM3 and CCCS3.

Figure 3a,b show the TPCDs and Fisher criteria for the CCSM3 and CCCS3 for all investigated harmonic sets using resonance frequencies $\omega_{c}$ of 14 and 30 Hz, without fault and with relative fault severities of 5% and 10% introduced for the defective cases.

The superiority of the CCSM3 over the CCCS3 can be seen in Figure 3a, with the CCSM3 having the total probability of correct diagnosis as high as 98% and as low as 92% and with the CCCS3 having 52% as the highest value of the total probability of correct diagnosis.

The Fisher criteria also validated the higher performance of the CCSM3 over the CCCS3 for all investigated cases. The CCSM3′s Fisher criterion result range was 3–8, as shown in Figure 3b, while that of CCCS3 was less than 1 for all investigated cases.

The simulation results generated from processing the modeled bilinear signals with resonance frequencies of 14 and 30 Hz, with and without damage at relative severities of 5% and 10%, and with the addition of white Gaussian noise demonstrated that the CCSM3 technique was highly effective in detecting nonlinearities resulting from component faults. The performance of the CCSM3 technology was compared to the performance of the CCCS3 technology using the TPCDs and Fisher criteria, which confirmed the superiority of the CCSM3 in diagnosing early-stage faults. Therefore, the CCSM3 technology was an efficient technique for identifying incipient faults.

### 3.2. Validation of Fourth Order Cross-Correlations via Modeling

The CCSM4 and CCCS4 are functions of four independent frequencies. The CCSM4 and CCCS4 were estimated using the following harmonic sets: (1,1,2,3), (1,2,4,5), (1,2,3,4) and (1,3,4,5). The fourth cross-correlations for harmonic set (1,1,2,3) were estimated for the four resonance harmonics: the fundamental resonance harmonic employed twice, the second resonance harmonic, and the third resonance harmonic. The triple cross-correlations for harmonic set (1,2,4,5) were estimated for the four resonance harmonics: the fundamental resonance harmonic, the second resonance harmonic, the fourth resonance harmonic, and the fifth resonance harmonic. The fourth cross-correlations for harmonic set (1,2,3,4) were estimated for the four resonance harmonics: the fundamental resonance harmonic, the second resonance harmonic, the third resonance harmonic, and the fourth resonance harmonic. Finally, the fourth cross-correlations for harmonic set (1,3,4,5) were estimated for the four resonance harmonics: the fundamental resonance harmonic, the third resonance harmonic, the fourth resonance harmonic, and the fifth resonance harmonic. These sets of resonance harmonics were investigated using resonance frequencies $\omega_{c}$ of 14 and 30 Hz for components without damage and with relative damage severities of 5% and 10% introduced for the damaged cases.

**Figure 4:**
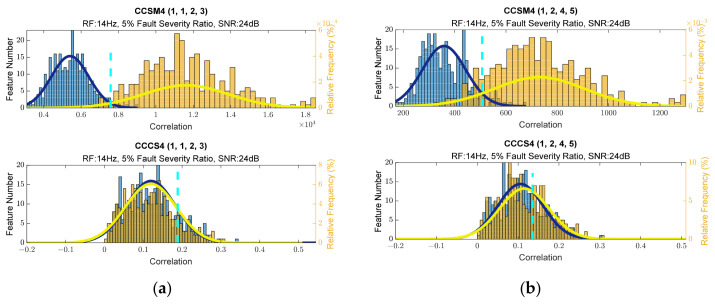
(**a**) Histograms of diagnostic features for (1,1,2,3) harmonic set. (**b**) Histograms of diagnostic features for (1,2,4,5) harmonic set.

To estimate the CCSM4 and CCCS4, SNR of 24 dB was used for 5% relative damage severity and SNR of 19 dB was used for 10% relative damage severity for resonance frequencies of 14 and 30 Hz Figure 4a,b show histograms for the selected diagnostic features. The histograms for additional frequency sets, taken into consideration for the CCSM4 and CCCS4, are presented in Figure A40, Figure A41, Figure A42 and Figure A43 in Appendix A. The superiority of the CCSM4 over the CCCS4 was also observed on these histograms.

**Figure 5:**
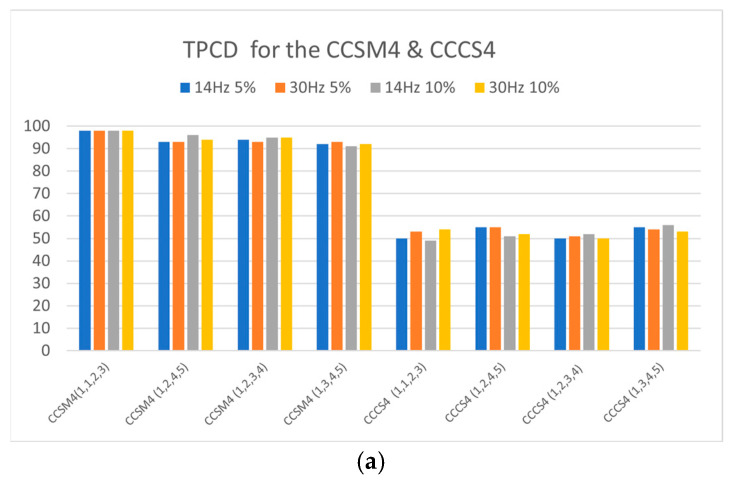

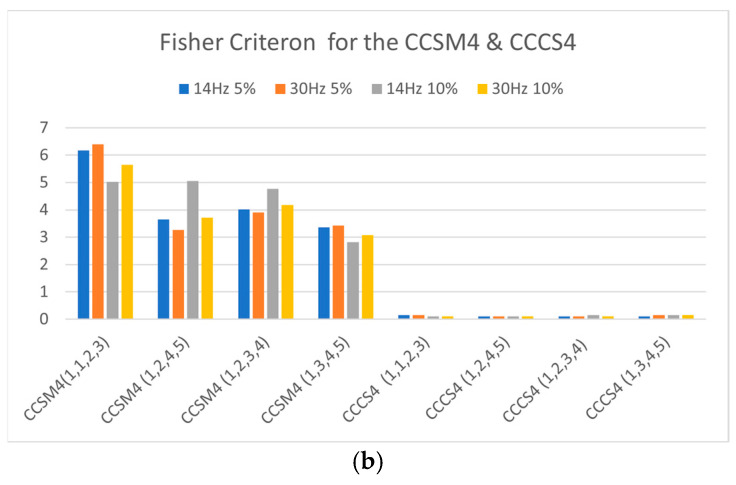
(**a**) The TPCDs for all investigated cases for the CCSM4 and CCCS4. (**b**) The Fisher criteria for all investigated cases for the CCSM4 and CCCS4.

Figure 5a,b show the TPCDs and Fisher criteria for the CCSM4 and CCCS4 for all investigated harmonic sets for resonance frequencies $\omega_{c}$ of 14 and 30 Hz without damage and for relative damage severities of 5% and 10%.

The superiority of the CCSM4 over the CCCS4 is shown in Figure 5a, with the CCSM4 having a TPCD as high as 98% and as low as 92%, while the CCCS4 had 56% as the highest value of the TPCD. The Fisher criteria also validated the higher performance of the CCSM4 over the CCCS4 for all investigated cases. The CCSM4′s Fisher criteria range was 3.0–6.4, as shown in Figure 5b, while for the CCCS4, it was less than 1 for all investigated cases.

The simulation results acquired by processing the modeled bilinear components with resonance frequencies of 14 and 30 Hz, at fault severity ratios of 5% and 10%, and in the presence of white Gaussian noise suggested that the CCSM4 technology was proficient in detecting nonlinearities resulting from component faults. The TPCDs and Fisher criteria were utilized to compare the performance of the CCSM4 to the CCCS4, revealing the superior diagnostic capability of the CCSM4 in identifying early-stage faults. Therefore, the CCSM4 technology was an efficient technique for diagnosing incipient faults.

## 4. Experimental Technology Validation

**Figure 6:**
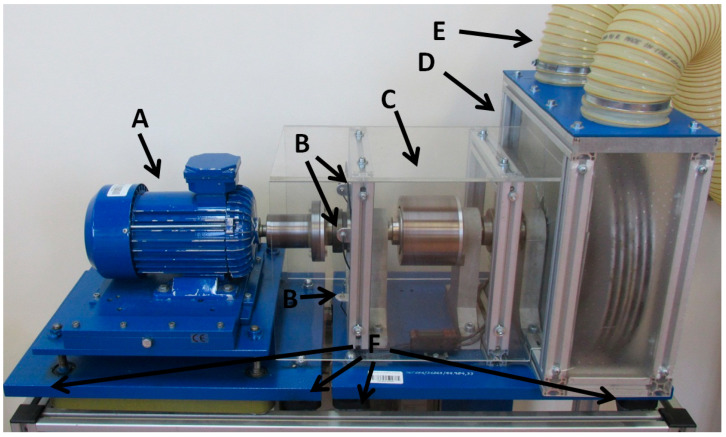
Experimental test rig: arrow A directs to the motor, the B arrows direct to the laser sensors, arrow C directs to the magnetic coupling, arrow D directs to the electromagnetic brake, arrow E directs to the cooling hoses, and the F arrows directs to the vibration isolation pads.

The laboratory test rig used in this research is described in detail in ref. [1,2]. A photograph of the test rig is presented in Figure 6. The IM used in this experiment was a three-phase 1.1 kW motor with two pole pairs. The tested IM was supplied directly from the electrical power grid.

**Figure 7:**
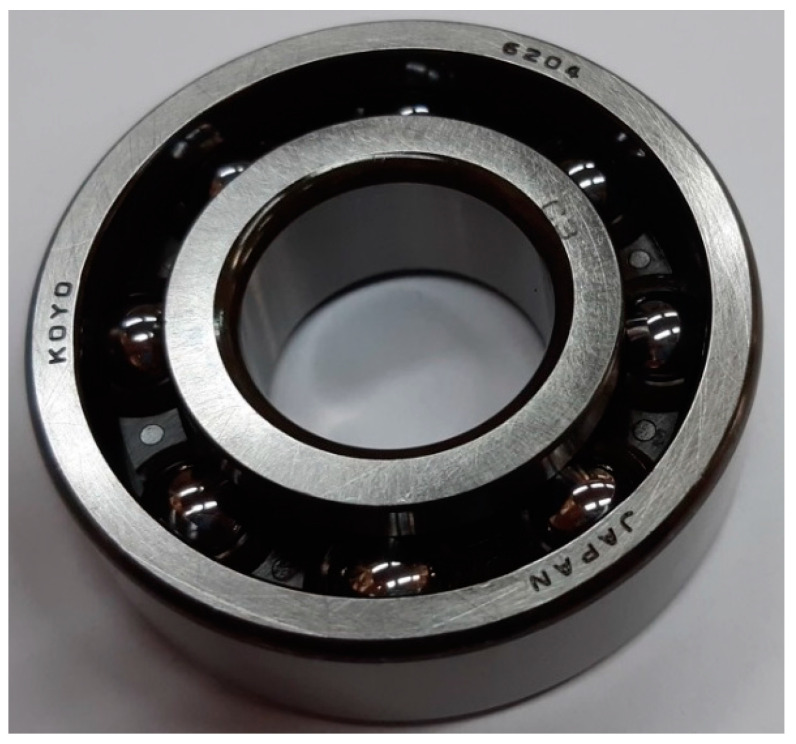
New Koyo 6204C3 bearing.

**Figure 8:**
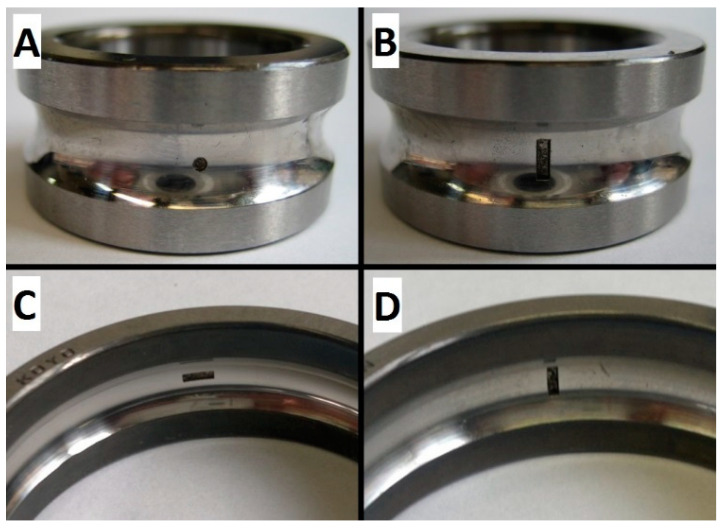
Close-up photographs of the introduced damage. (**A**) bearing in1, (**B**) bearing in2, (**C**) bearing out1, (**D**) bearing out2.

All measurements used in this experiment were made under the conditions of a full motor load. All experimental tests were performed with 6204C3 Koyo bearings. One of the new tested bearings is shown in Figure 7. The IM current recordings were performed for 4 new bearings and 4 defective bearings with intentionally made damage of different types and severity. Close-up photographs of the introduced damage are presented in Figure 8. The description of each damage is presented in Table 1. All introduced defects were local faults to reproduce conditions of early-stage bearing defect development. The new bearings used in this experiment are described as p1, p2, p3, and p4.

**Figure 9:**
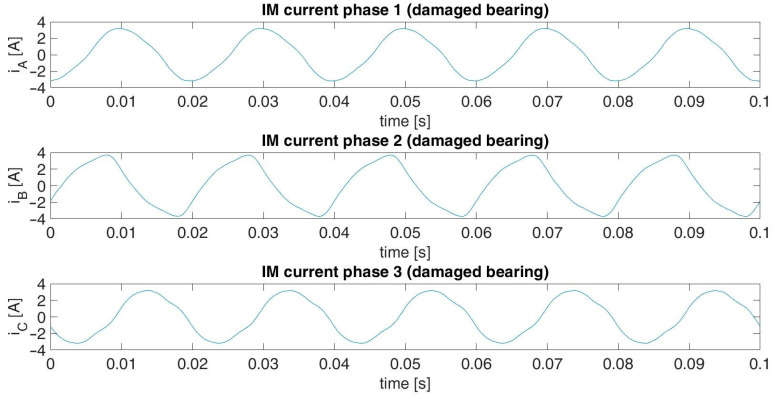
Fragment of the captured IM current signals for a damaged bearing.

**Figure 10:**
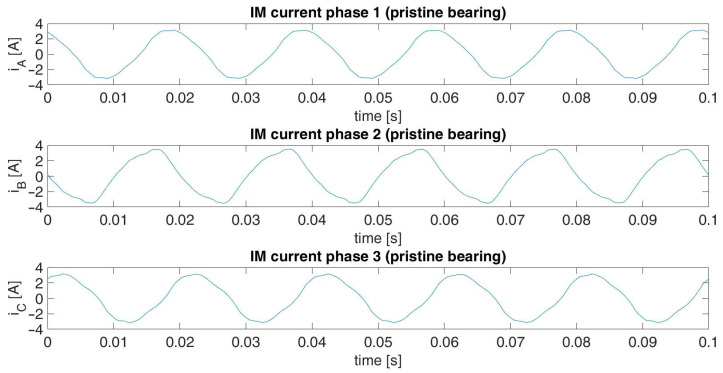
Fragment of the captured IM current signals for a pristine bearing.

The acquired data for each diagnostic case included three digital standard current signals of three phases of the induction motor. These signals were not normalized. The length of each signal was 65 s acquired with a sampling frequency of 65 kHz. The current signals were captured for stationary motor operation under full load conditions. The main component of each signal was the 50 Hz supply current component. Graphical illustrations of fragments of the recorded signals for the damaged and pristine bearings are presented in Figure 9 and Figure 10, respectively.

The captured signals were split into 13 non-overlapping time segments of 5 s. The Blackman time window was used for each segment of the signal. The frequency of the IM shaft rotation $f_{r}$ and the frequency of the power supply $f_{p}$ were estimated from the IM current spectrum for each time segment separately. It is crucial in the examined technologies to track changes in the aforementioned frequencies in each time segment in order to track the bearing defect frequencies. The values of the IM shaft rotation frequency $f_{r}$ were estimated using the detection of rotor slot harmonics (*RSH*) frequency and the parameters of the used IM constructions [46,76]. After detection of the *RSH* frequency, Equation (14) was used for each $f_{r}$ estimation [46,76]:

(14) $$f_{r}=\frac{f_{RSH}\pm f_{p}}{k\cdot N_{R}}$$

where $f_{RSH}$ is the frequency of *RSH*, $f_{p}$ is the frequency of the power supply, $f_{r}$ is the IM shaft rotation frequency, k is the number of rotor bar harmonics, and $N_{R}$ is the number of rotor bars.

The values of these frequencies are needed to estimate the characteristic frequencies related to bearing damage. Next, the complex spectral amplitude for the CCCS3 and the modulus of the complex spectral amplitude for the CCSM3 from the current spectra were obtained for three bearing defect-related characteristic frequencies for each segment. The following step is to use Equation (2) for the CCSM3 estimation and Equation (5) for the CCCS3 estimation. The final step is to calculate the mean values of the CCSM3 and CCCS3 for three phases of the captured IM current. The values of the CCSM3 and CCCS3, obtained via the described algorithm are used as diagnostic features for IM bearing diagnostics and for the comparison of the CCSM3 and CCCS3.

The bearing defect frequencies are described by the following equations [1,2,19]:

(15) $$f_{out}=\frac{1}{2}f_{r}\left(1-\frac{B_{d}}{P_{d}}\cos\alpha \right)N_{b}={FCC}_{o}\cdot f_{r}$$

(16) $$f_{in}=\frac{1}{2}f_{r}\left(1+\frac{B_{d}}{P_{d}}\cos\alpha \right)N_{b}={FCC}_{i}\cdot f_{r}$$

where $f_{out}$ is the defect frequency for the outer ring, $f_{in}$ is the defect frequency for the inner ring, $f_{r}$ is the shaft rotation frequency, $N_{b}$ is the number of balls or rollers, $B_{d}$ is the diameter of the balls or rollers, $P_{d}$ is the pitch diameter of a bearing, and α is the angle of thrust. ${FCC}_{o}$ is the fault characteristic coefficient for the outer race, while ${FCC}_{i}$ is the fault characteristic coefficient for the inner race. Coefficients ${FCC}_{i}$ and ${FCC}_{o}$ are provided by SKF.

To compare the CCSM3 with the CCCS3, 51 diagnostic features for each bearing were calculated for each examined technology. Time segments overlapping from 0% to 50% with 1% steps were used for these calculations. The values of 102 diagnostic features were used for estimations of the TPCD and FC. IM current signals were recorded for four new bearings and four defective bearings. The four pairs (new–defective) of bearings were split into two cases: two bearings with outer race defects and two bearings with inner race defects. Each pair of bearings was examined separately and the obtained results were averaged. The recorded signals were processed via the examined CCSM3 and CCCS3 technologies in order to make a comparison.

In the current stage, the acquired data were processed on a separate computer via Matlab R2021a. The computational/measurement complexity are discussed below. All data in the experimental part were processed on PC with Intel Core i7-2600 CPU and 8 GB of RAM (by Intel Corporation, Santa Clara, CA, USA). The computation time on this hardware acquired with the Matlab cpu time command was 1.9812 s for 0% overlapping and 3.3696 s for 50% overlapping. It took 123.5 s to calculate 51 diagnostic features. If the CCSM3 algorithm software was organized, using the calculation of the CCSM3 during the sample intervals, then the CCSM3 feature could be estimated in “real time.” The signals were the three phases of the motor current, the length was 65 s, the sampling frequency was 65,536 Hz, and 24 bits were used for the sampling.

The total number of operations acquired with Matlab socFunctionAnalyzer (by MathWorks, Natick, MA, USA) was 310,778,788 for the original 65,536 Hz sampling frequency, 65 s of signal duration, and 5 s duration of time segments with no overlapping. The number of operations could be significantly decreased by decreasing the sampling frequency used in this experiment. After downsampling the signal by 2 (with the same all other parameters) the total number of operations was decreased to 155,458,468. The total number of operations for further downsampling is presented in the Table 2.

Based on the theoretical basis, the number of operations can be estimated as follows.

Considering a conventional method of CCSM3 estimation, the main method for estimation of the CCSM3 is the direct method. Extension of this method to the CCSM4 or to any higher CCSM order can be made easily. Considering the direct method of estimation of the numerator of the CCSM3 for one current phase (since estimation of the denominator of the CCSM3 is a computationally easy task) for one diagnostic feature, the following steps should be employed:

- segment current data into K non-overlapped segments of N samples each
- estimate DFT coefficients for each segment
- calculate CCSM3 estimates for all segments via multiplications of moduli of the selected DFT coefficients and average these estimates over K segments

The number of multiplication operations to estimate the DFT for each segment is $N\log_{2}N$, and the number of addition operations to estimate the DFT for each segment is $2N\log_{2}N$.

Finally, ignoring a small number of addition operations for the averaging of the CCSM3 over K segments and a small number of multiplication operations for obtaining the CCSM3 for each segment, the total number of multiplication operations for K segments is K × $N\log_{2}N$ and total number of addition operations for K segments is K × $N\log_{2}N$.

Using the above methodology to estimate the total number of operations for calculation of the numerator of the CCSM3, the numbers of multiplication and addition operations were 78,040,269 and 156,080,538, respectively, for the processing parameters employed in the experimental trials, i.e., the segment size was 5 s, the number of segments was 13, and the sampling frequency was 65.536 kHz with 0% segment overlapping. Therefore, the total number of operations (i.e., additions and multiplications) for calculation of the numerator of the CCSM3 was 234,120,807.

### 4.1. Inner Race Defect Analysis

Components with the frequency related to bearing inner race defects, described by Equation (16), did not directly appear in the IM current spectrum. The frequencies of the components of the IM current related to bearing inner race defects can be described by Equation (17) [28,36]:

(17) $$f_{comp\_in}={i\cdot f}_{in}+j\cdot f_{p}+k\cdot f_{r}$$

where $f_{in}$ is the inner race defect frequency, $f_{p}$ is the frequency of the power supply, $f_{r}$ is the frequency of the IM shaft rotation, and i,j,k are integer coefficients related to each frequency.

The bearings used in this part of experiment were the pairs of bearings in1, p1 and in2, p2. The results obtained in this part were estimated with the use of 84 components, which were used to calculate the CCSM3 and CCCS3 for 28 different combinations of three components for each bearing. The 84 components were obtained with the i,j,k coefficients [1]:

i=3,6,9;j=−3,−1,1,3;k=−3,−2,−1,0,1,2,3.

**Figure 11:**
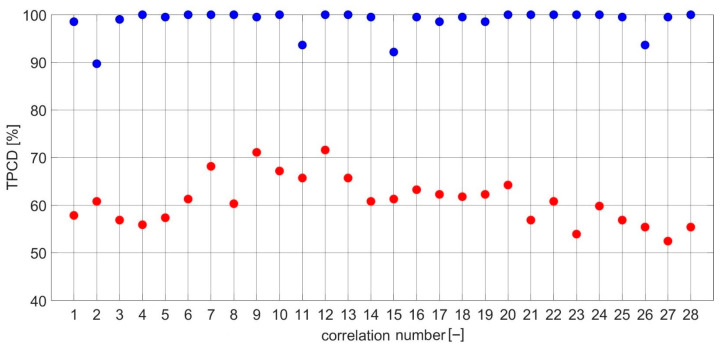
Graph of the probability of proper inner race diagnosis with the CCSM3 (**blue**) and CCCS3 (**red**) for each correlation number (Table A1).

**Figure 12:**
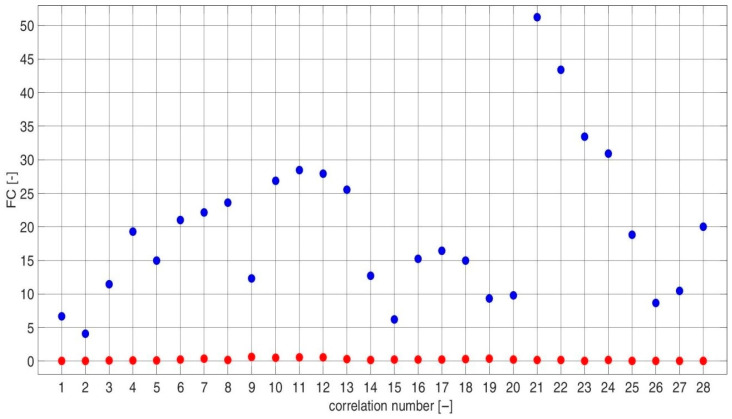
Value of the Fisher criterion calculated for the CCSM3 (**blue**) and CCCS3 (**red**) for each correlation number (Table A1).

**Figure A1:**
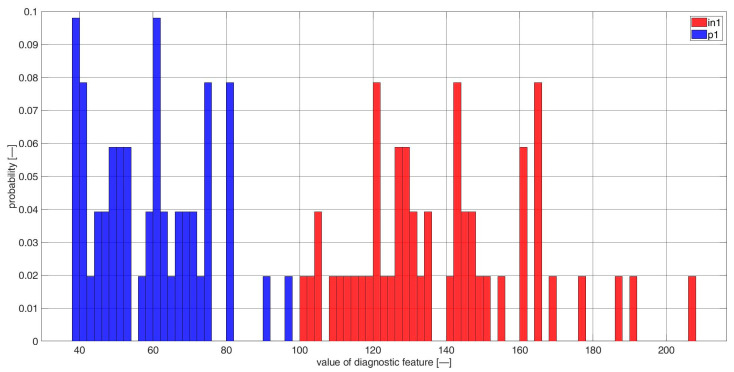
Histogram of diagnostic features based on the CCSM3 (correlation number is 4—Table A1), bearings in1 and p1.

**Figure A2:**
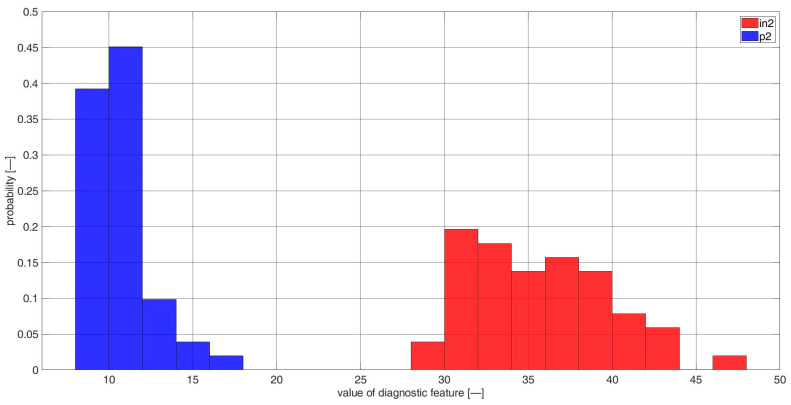
Histogram of diagnostic features based on the CCSM3 (correlation number is 4—Table A1), bearings in2 and p2.

**Figure A3:**
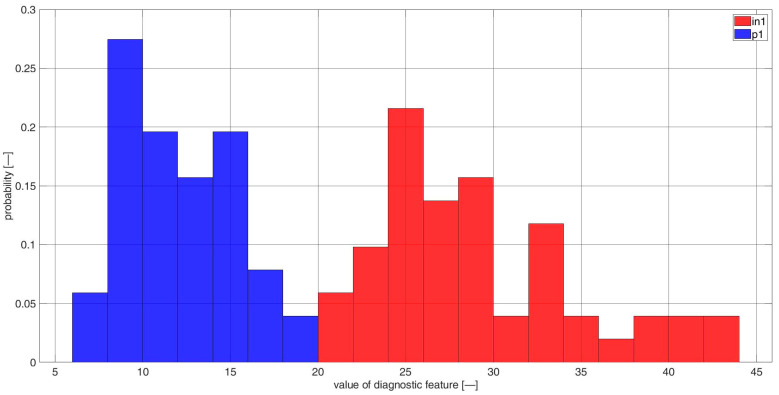
Histogram of diagnostic features based on the CCSM3 (correlation number is 5—Table A1), bearings in1 and p1.

**Figure A4:**
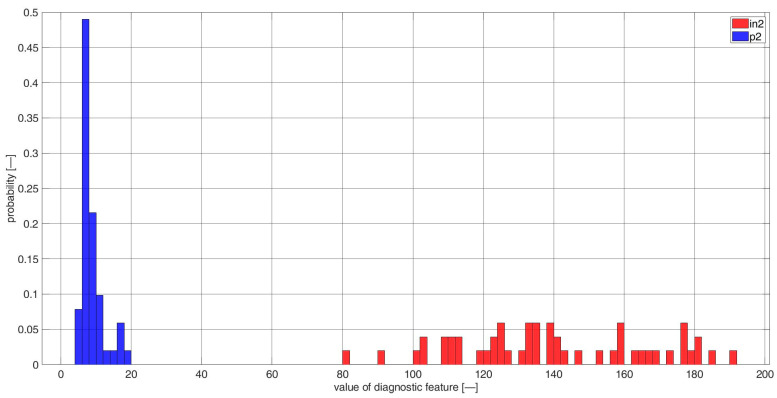
Histogram of diagnostic features based on the CCSM3 (correlation number is 5—Table A1), bearings in2 and p2.

**Figure A5:**
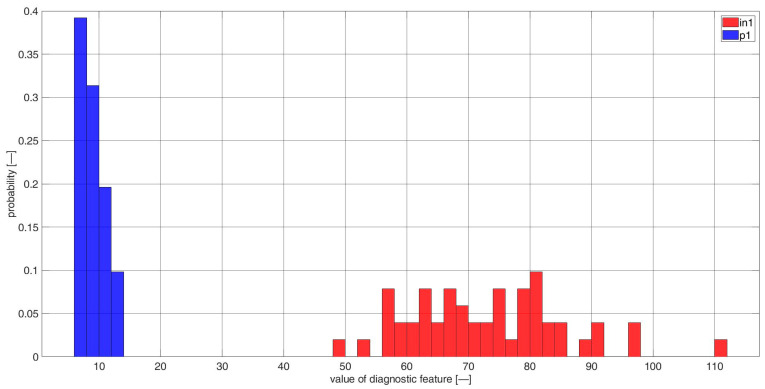
Histogram of diagnostic features based on the CCSM3 (correlation number is 6—Table A1), bearings in1 and p1.

**Figure A6:**
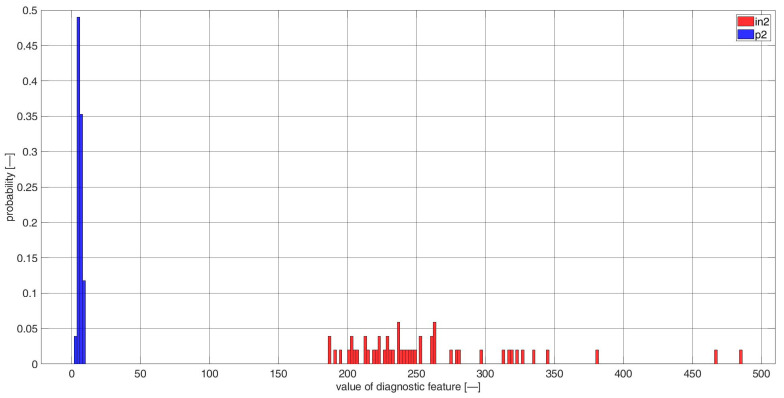
Histogram of diagnostic features based on the CCSM3 (correlation number is 6—Table A1), bearings in2 and p2.

**Figure A7:**
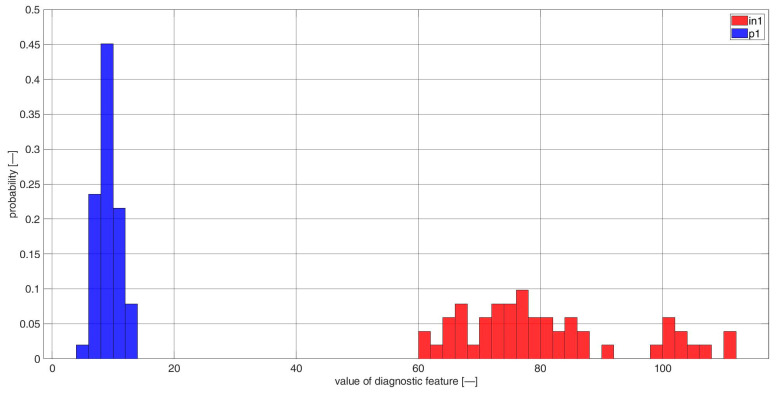
Histogram of diagnostic features based on the CCSM3 (correlation number is 7—Table A1), bearings in1 and p1.

**Figure A8:**
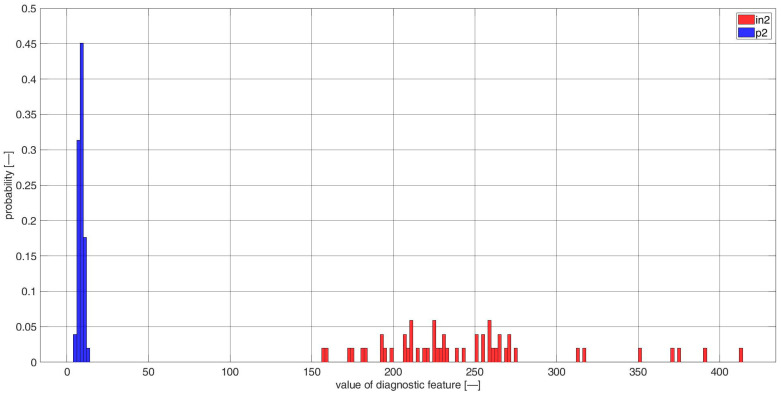
Histogram of diagnostic features based on the CCSM3 (correlation number is 7—Table A1), bearings in2 and p2.

**Figure A9:**
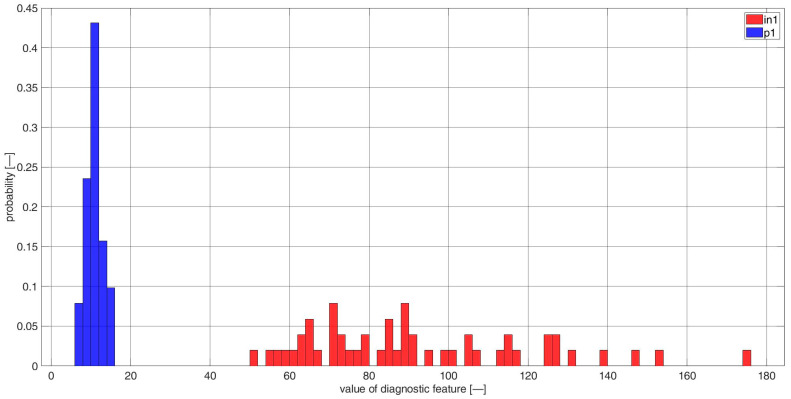
Histogram of diagnostic features based on the CCSM3 (correlation number is 8—Table A1), bearings in1 and p1.

**Figure A10:**
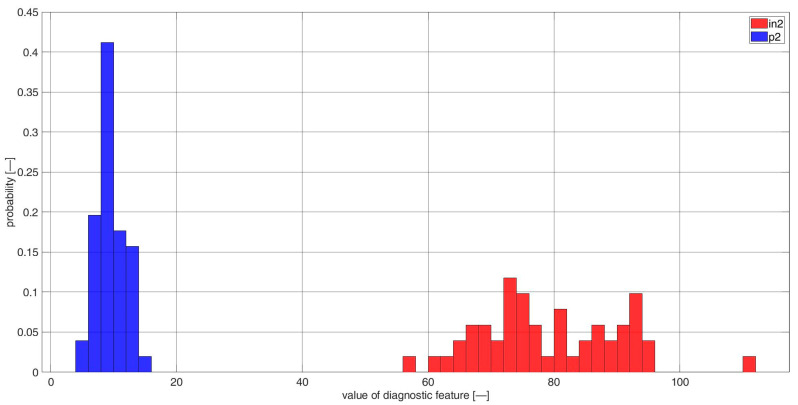
Histogram of diagnostic features based on the CCSM3 (correlation number is 8—Table A1), bearings in2 and p2.

**Figure A11:**
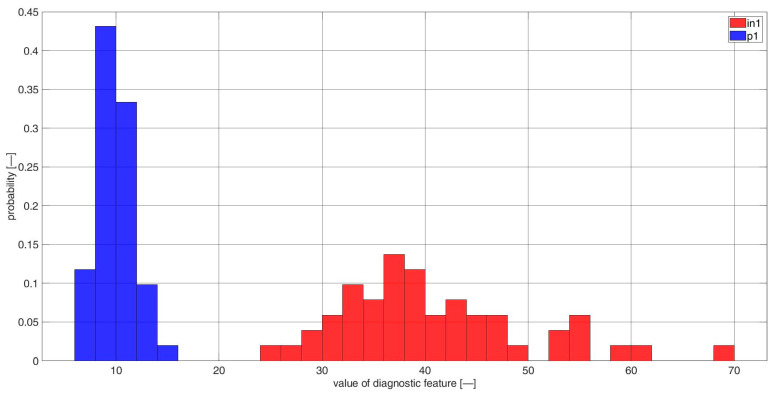
Histogram of diagnostic features based on the CCSM3 (correlation number is 10—Table A1), bearings in1 and p1.

**Figure A12:**
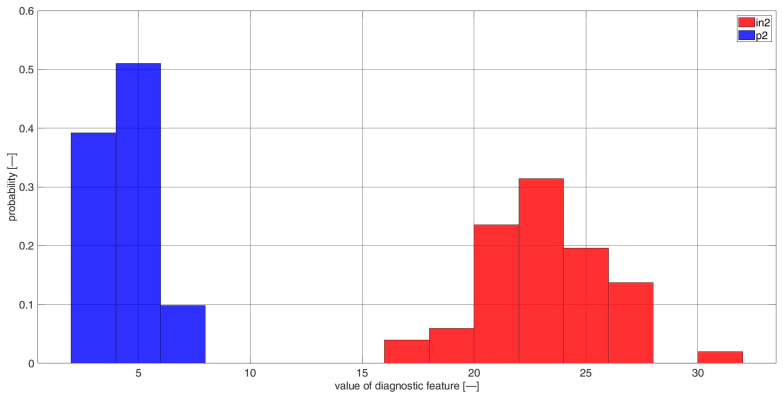
Histogram of diagnostic features based on the CCSM3 (correlation number is 10—Table A1), bearings in2 and p2.

**Figure A13:**
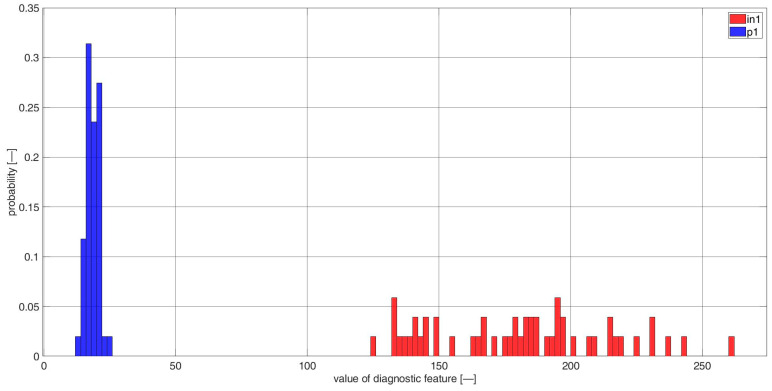
Histogram of diagnostic features based on the CCSM3 (correlation number is 12—Table A1), bearings in1 and p1.

**Figure A14:**
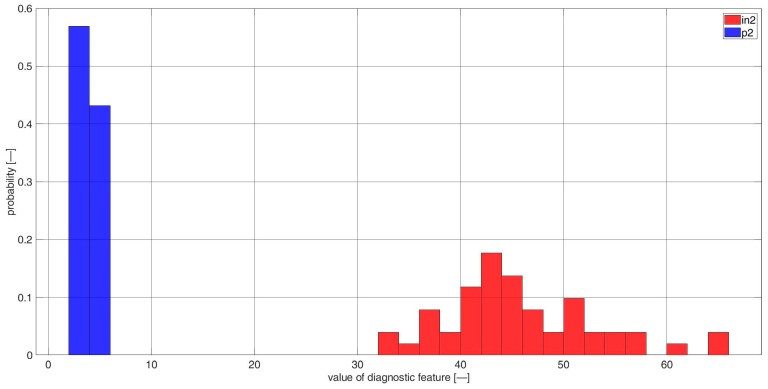
Histogram of diagnostic features based on the CCSM3 (correlation number is 12—Table A1), bearings in2 and p2.

**Figure A15:**
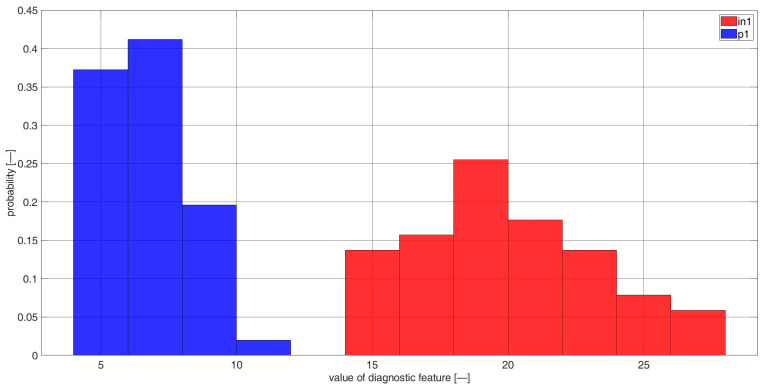
Histogram of diagnostic features based on the CCSM3 (correlation number is 13—Table A1), bearings in1 and p1.

**Figure A16:**
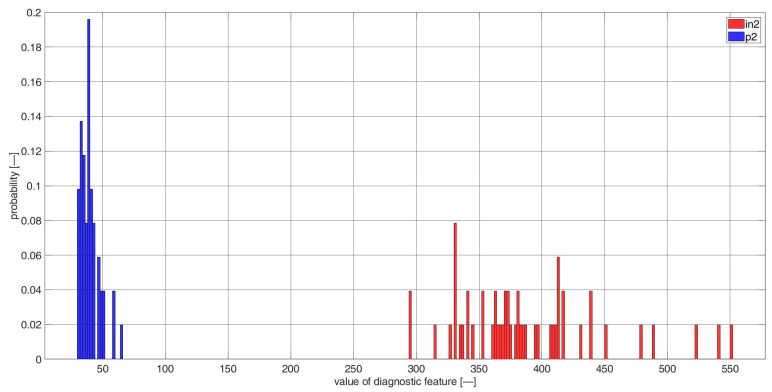
Histogram of diagnostic features based on the CCSM3 (correlation number is 13—Table A1), bearings in2 and p2.

**Figure A17:**
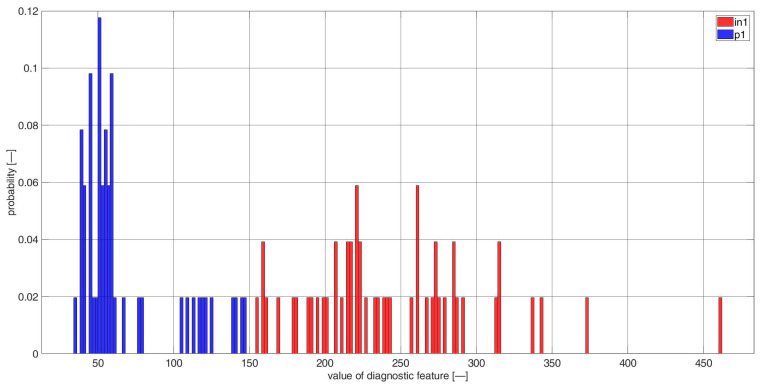
Histogram of diagnostic features based on the CCSM3 (correlation number is 20—Table A1), bearings in1 and p1.

**Figure A18:**
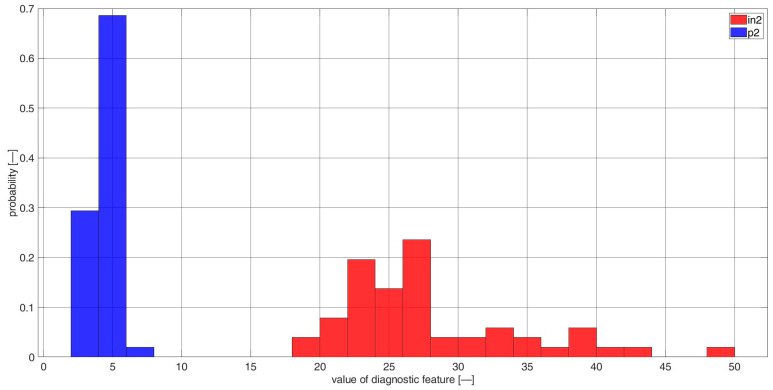
Histogram of diagnostic features based on the CCSM3 (correlation number is 20—Table A1), bearings in2 and p2.

**Figure A19:**
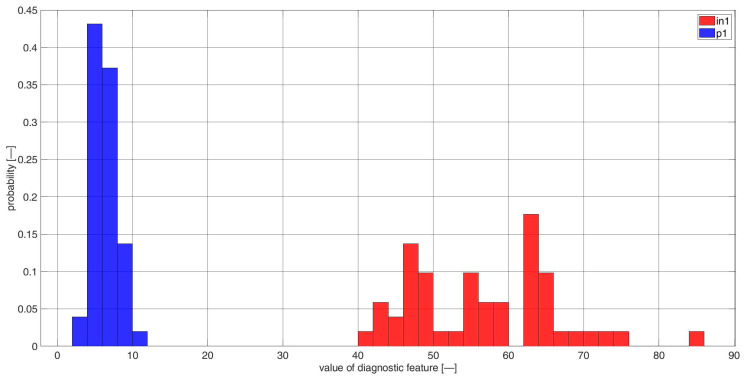
Histogram of diagnostic features based on the CCSM3 (correlation number is 21—Table A1), bearings in1 and p1.

**Figure A20:**
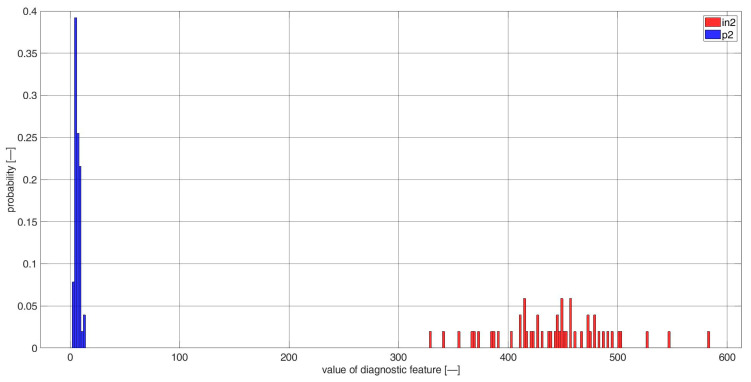
Histogram of diagnostic features based on the CCSM3 (correlation number is 21—Table A1), bearings in2 and p2.

**Figure A21:**
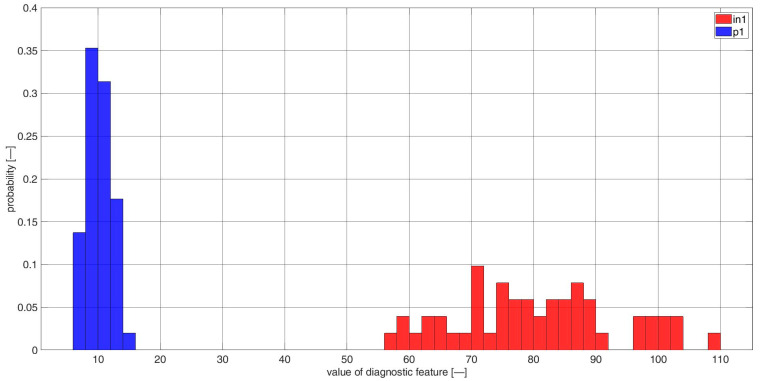
Histogram of diagnostic features based on the CCSM3 (correlation number is 22—Table A1), bearings in1 and p1.

**Figure A22:**
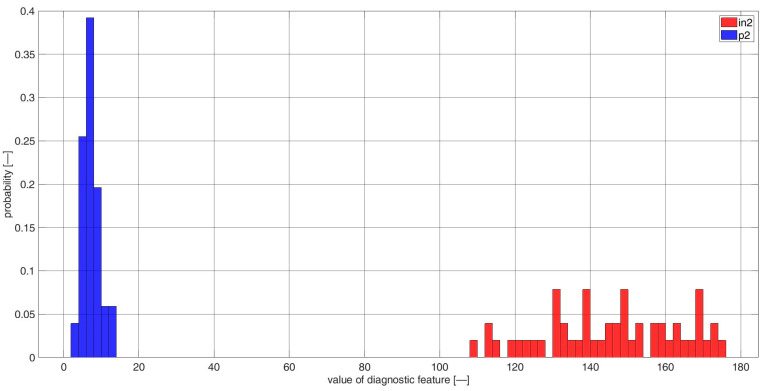
Histogram of diagnostic features based on the CCSM3 (correlation number is 22—Table A1), bearings in2 and p2.

**Figure A23:**
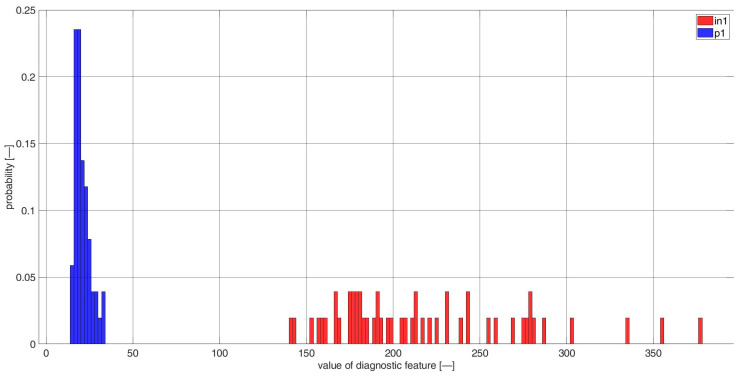
Histogram of diagnostic features based on the CCSM3 (correlation number is 23—Table A1), bearings in1 and p1.

**Figure A24:**
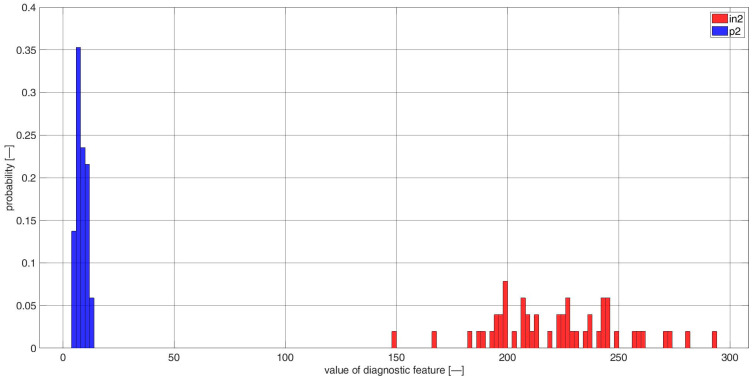
Histogram of diagnostic features based on the CCSM3 (correlation number is 23—Table A1), bearings in2 and p2.

**Figure A25:**
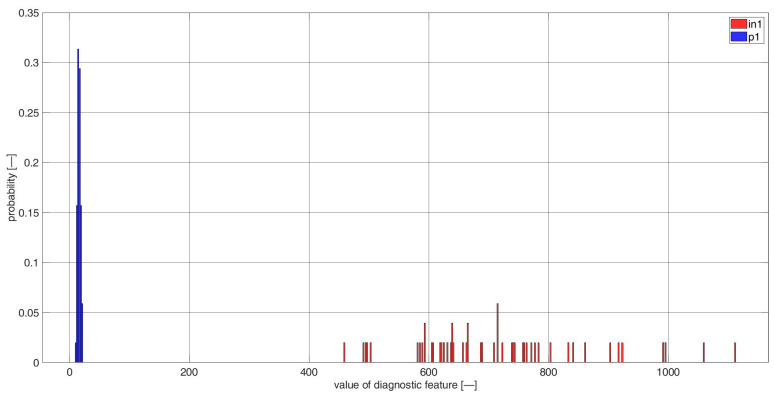
Histogram of diagnostic features based on the CCSM3 (correlation number is 24—Table A1), bearings in1 and p1.

**Figure A26:**
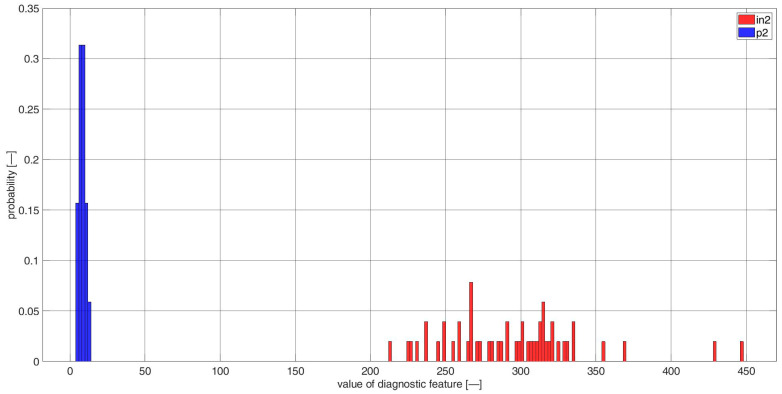
Histogram of diagnostic features based on the CCSM3 (correlation number is 24—Table A1), bearings in2 and p2.

**Figure A27:**
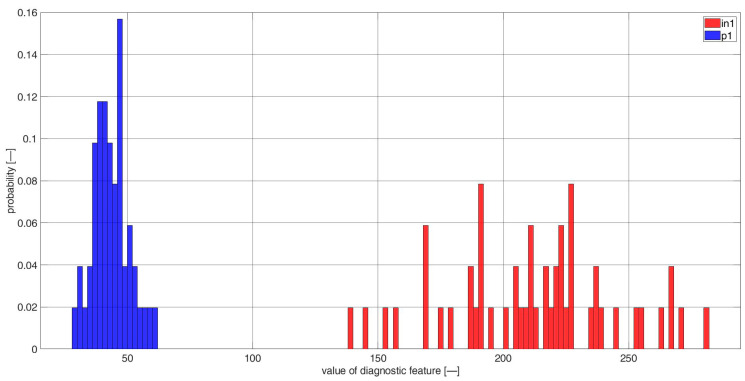
Histogram of diagnostic features based on the CCSM3 (correlation number is 28—Table A1), bearings in1 and p1.

**Figure A28:**
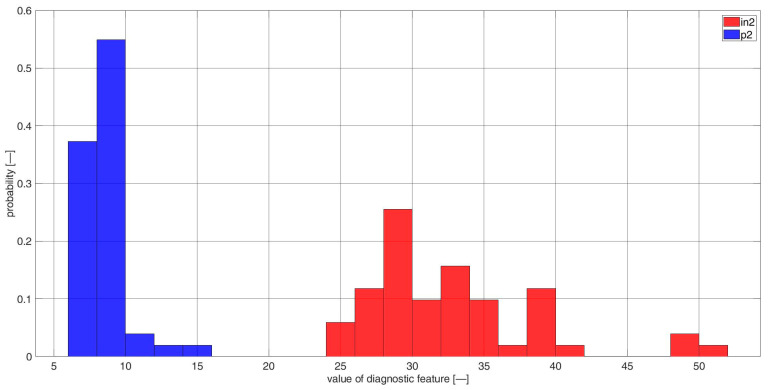
Histogram of diagnostic features based on the CCSM3 (correlation number is 28—Table A1), bearings in2 and p2.

For each i, different sets of j and k coefficients were used. The values of each diagnostic feature based on the CCSM3 and CCCS3 were estimated for three individual combinations of i,j,k coefficients. The values of the i,j,k coefficients corresponding with each number of the CCSM3 and CCCS3 correlation are listed in Table A2 in Appendix A. TPCDs, estimated via Equation (7) for the CCSM3 and CCCS3 technologies, are presented in Figure 11 The average TPCD, calculated for all 28 correlations, was 99% for the CCSM3 and 61% for the CCCS3. For the same data, the Fisher criteria were estimated. The values of the Fisher criteria for each correlation number (Table A1) are presented in Figure 12. The average Fisher criterion values were 19.5 for the CCSM3 and 0.2 for the CCCS3. Histograms of the diagnostic features values based on the CCSM3 are presented in Figure A1, Figure A2, Figure A3, Figure A4, Figure A5, Figure A6, Figure A7, Figure A8, Figure A9, Figure A10, Figure A11, Figure A12, Figure A13, Figure A14, Figure A15, Figure A16, Figure A17, Figure A18, Figure A19, Figure A20, Figure A21, Figure A22, Figure A23, Figure A24, Figure A25, Figure A26, Figure A27 and Figure A28 in Appendix A. Each pair of presented histograms displayed full separation between the cases of new and defective bearings.

### 4.2. Outer Race

Components with the frequency related to bearing outer race defects described by Equation (14) did not directly appear in the IM current spectrum. The frequencies of the components of the IM current related to bearing outer race defects can be described by Equation (17) [5,28,36]:

(18) $$f_{comp\_out}={i\cdot f}_{out}+j\cdot f_{p}$$

where $f_{out}$ is the outer race defect frequency, $f_{p}$ is the frequency of the power supply, and i,j are the integer coefficients related to each frequency.

**Figure 13:**
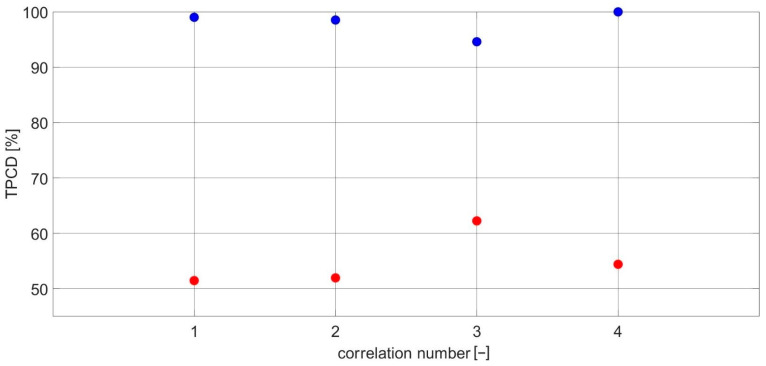
The complete probability of correct outer race diagnosis with the CCSM3 (**blue**) and CCCS3 (**red**) for each correlation number (Table A2).

**Figure 14:**
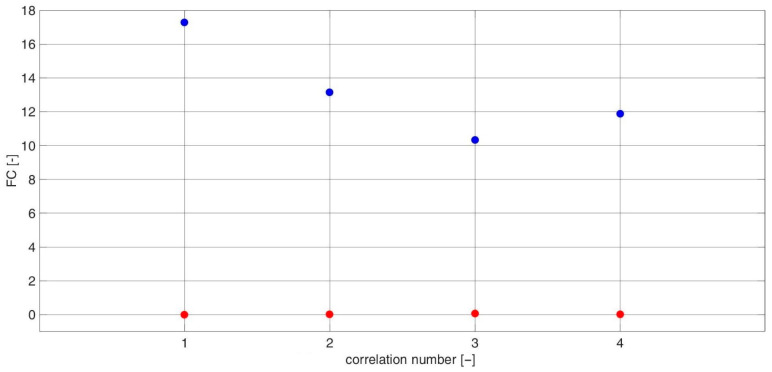
Value of the Fisher criterion calculated for the CCSM3 (**blue**) and CCCS3 (**red**) for each correlation number (Table A2).

**Figure A29:**
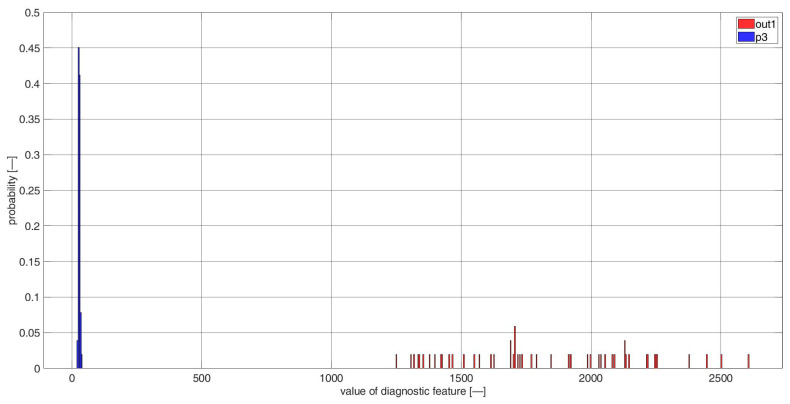
Histogram of diagnostic features based on the CCSM3 (correlation number is 1—Table A2), bearings out1 and p3.

**Figure A30:**
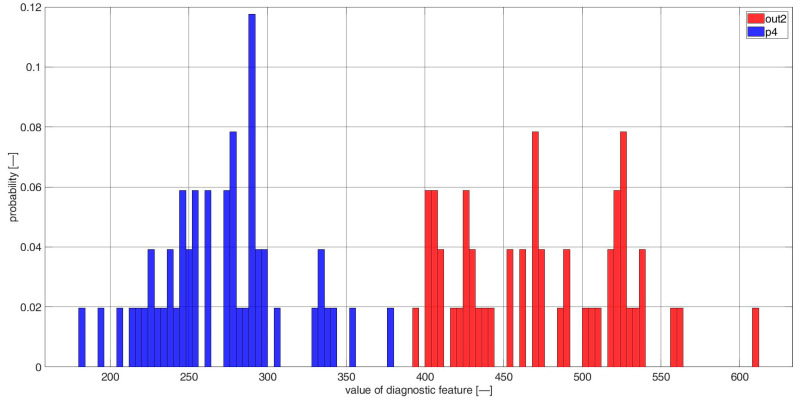
Histogram of diagnostic features based on the CCSM3 (correlation number is 1—Table A2), bearings out2 and p4.

**Figure A31:**
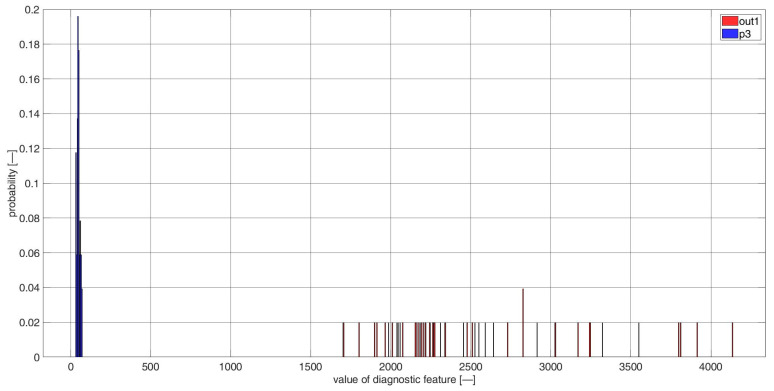
Histogram of diagnostic features based on the CCSM3 (correlation number is 2—Table A2), bearings out1 and p3.

**Figure A32:**
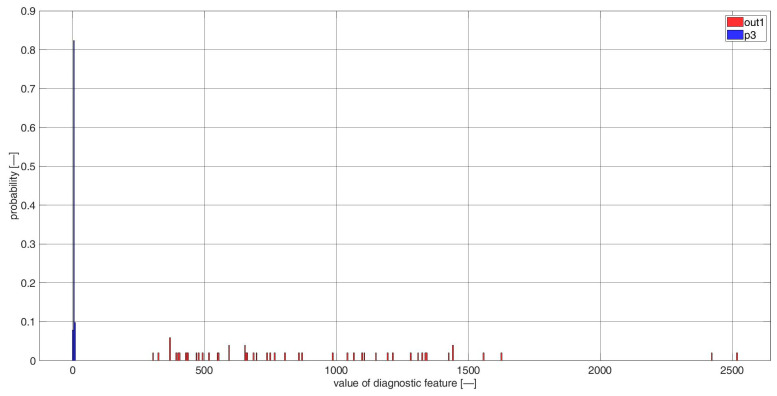
Histogram of diagnostic features based on the CCSM3 (correlation number is 3—Table A2), bearings out1 and p3.

**Figure A33:**
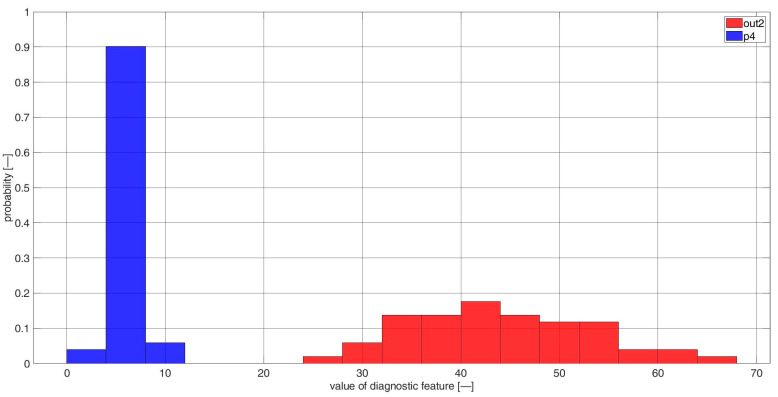
Histogram of diagnostic features based on the CCSM3 (correlation number is 3—Table A2), bearings out2 and p4.

**Figure A34:**
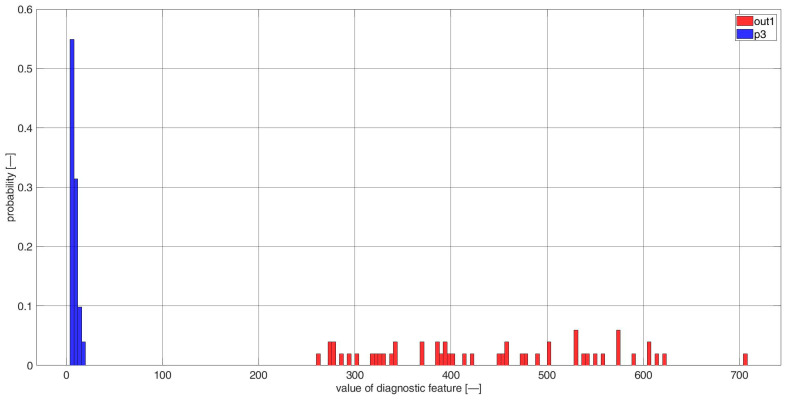
Histogram of diagnostic features based on the CCSM3 (correlation number is 4—Table A2), bearings out1 and p3.

**Figure A35:**
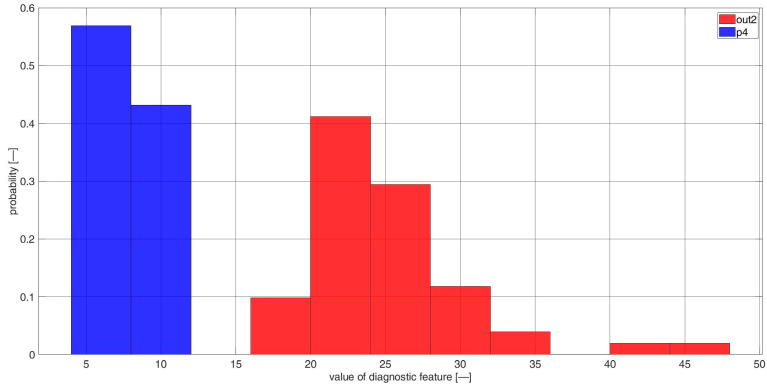
Histograms of diagnostic features based on the CCSM3 (correlation number is 4—Table A2), bearings out2 and p4.

**Figure A36:**
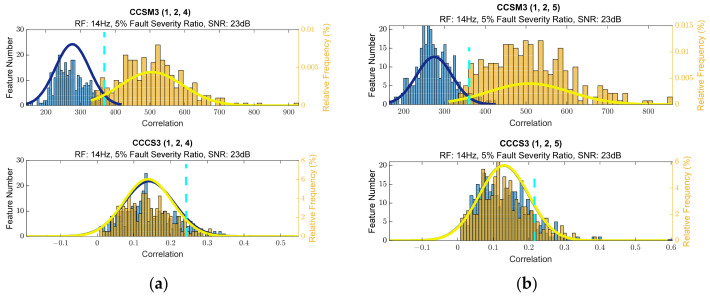
(**a**) Histograms of diagnostic features for (1,2,4) harmonics. (**b**) Histograms of diagnostic features for (1,2,5) harmonics.

**Figure A37:**
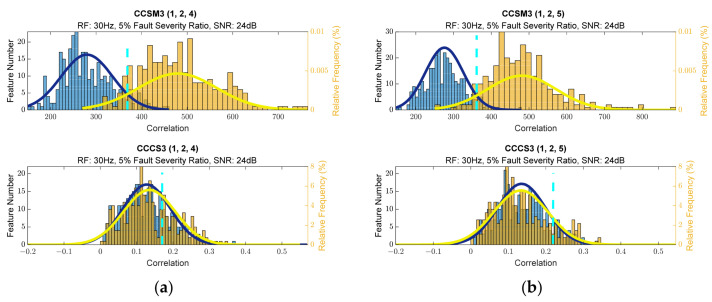
(**a**) Histograms of diagnostic features for (1,2,4) harmonics. (**b**) Histograms of diagnostic features for (1,2,5) harmonics.

**Figure A38:**
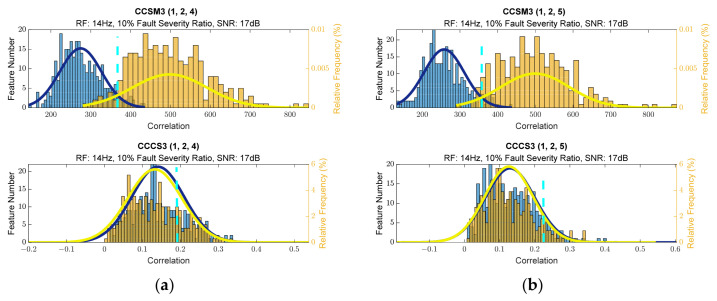
(**a**) Histograms of diagnostic features for (1,2,4) harmonics. (**b**) Histograms of diagnostic features for (1,2,5) harmonics.

**Figure A39:**
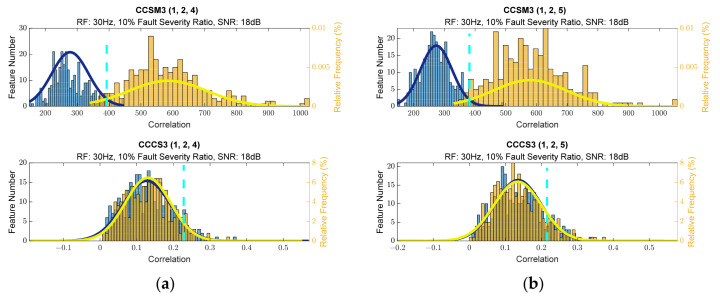
(**a**) Histograms of diagnostic features for (1,2,4) harmonics. (**b**) Histograms of diagnostic features for (1,2,5) harmonics.

**Figure A40:**
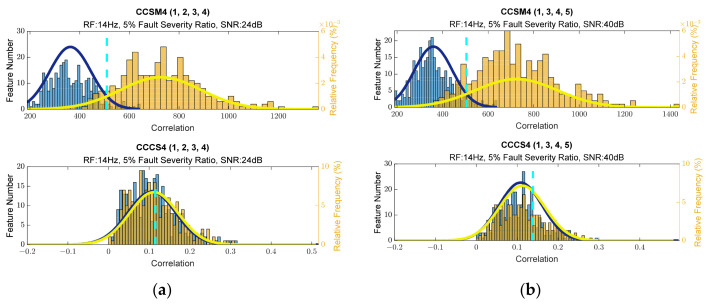
(**a**) Histograms of diagnostic features for (1,2,3,4) harmonics. (**b**) Histograms of diagnostic features for (1,3,4,5) harmonics.

**Figure A41:**
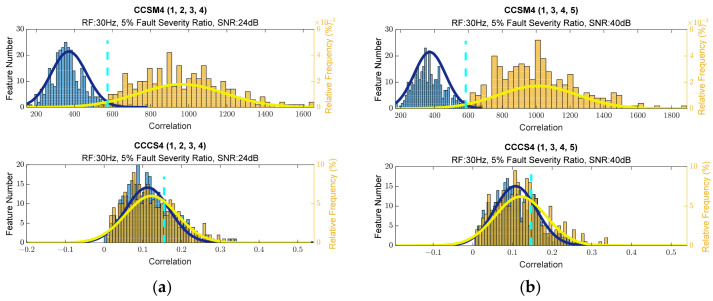
(**a**) Histograms of diagnostic features for (1,2,3,4) harmonics. (**b**) Histograms of diagnostic features for (1,3,4,5) harmonics.

**Figure A42:**
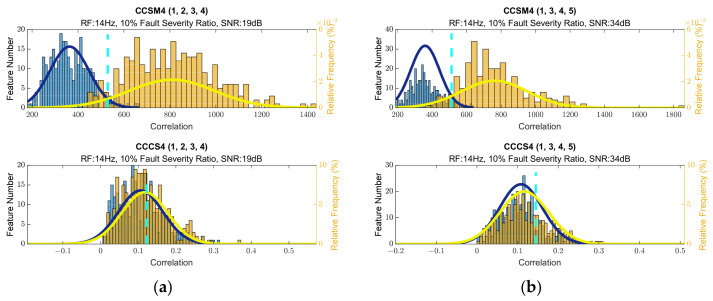
(**a**) Histograms of diagnostic features for (1,2,3,4) harmonics. (**b**) Histograms of diagnostic features for (1,3,4,5) harmonics.

**Figure A43:**
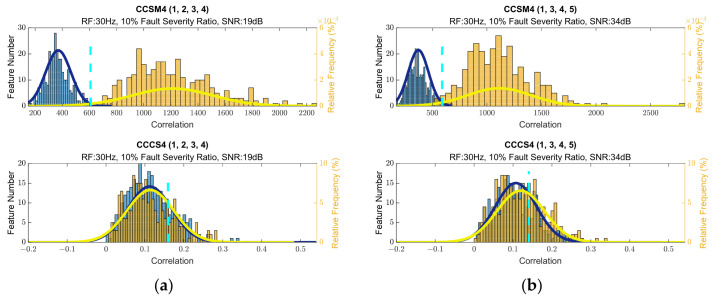
(**a**) Histograms of diagnostic features for (1,2,3,4) harmonics. (**b**) Histograms of diagnostic features for (1,3,4,5) harmonics.

The bearings used in this part of the experiment were the pairs of bearings out1, p3 and out2, p4. The results obtained in this part were estimated via the use of 12 components to calculate the CCSM3 and CCCS3 for 4 different combinations of three components for each bearing. The 12 components were obtained following the use of i,j coefficients [1]:

i=4,8;j=−5,−3,−1,1,3,5;

For each coefficient i, different sets of j coefficients were used. The values of each diagnostic feature based on the CCSM3 and CCCS3 were estimated for three individual combinations of the i,j coefficients. The values of the i,j coefficients corresponding with each number of the CCSM3 and CCCS3 correlations are listed in Table A2 in Appendix A. The TPCDs, estimated via Equation (7) for the CCSM3 and CCCS3 technologies are presented in the Figure 13. The average TPCD calculated for all 4 correlations was 98% for the CCSM3 and 55% for the CCCS3. For the same data, the Fisher criteria were estimated. The values of the Fisher criterion for each correlation number (Table A2) are presented in Figure 14. The average Fisher criterion values were 13.2 for the CCSM3 and 0.03 for the CCCS3. Histograms of the diagnostic features values based on the CCSM3 are presented in Figure A29, Figure A30, Figure A31, Figure A32, Figure A33, Figure A34 and Figure A35 in Appendix A. Each pair of presented histograms displayed full separation between the cases of new and defective bearings.

## 5. Technology Comparisons

The simulations and experimental results clearly showed that the CCSM3 was a more efficient signal processing technique than the CCCS3 for bearing fault diagnosis. The simulations were carried out for signal-to-noise ratios varying from 18 to 22 dB and fault severity ratios of 5% and 10%. The simulations included 4 different diagnosis features for two different resonance frequencies of 14 and 30 Hz for undamaged components. The experimental validation was carried out on 8 bearings: two with inner race damage, two with outer race damage, and 4 pristine bearings. For diagnostic purposes, the recorded motor current signals were processed. In the inner race experiments, 28 different diagnosis features were examined. In the outer race experiments, 4 different diagnosis features were examined.

A comparison of the achieved results is presented in Table 3. The gain of effectiveness for FC was calculated as the ratio of FC for the CCSM3 and for the CCCS3. The effectiveness gains for FC were 50.7 for the simulation results, 97.5 for the inner race experiment results, and 440 for the outer race experiment results. The gain in probability of incorrect diagnosis was calculated as the ratio of estimated probability of incorrect diagnosis for the CCCS3 and for the CCSM3. The effectiveness gains for the probability of incorrect diagnosis were 11.4 for the simulation results, 27.9 for the inner race experiment results, and 22.5 for the outer race experiment results. Both the simulation and experimental results confirmed that the CCSM3 method was a more efficient diagnosis method than the CCCS3 method.

The presented results clearly showed that the CCSM3 was an efficient method to detect mechanical damage in electromechanical systems. The simulations revealed that this new signal processing technique can perform well even with signals with low SNR. This feature is highly desirable especially for damage diagnosis with the use of electrical signals, such as a motor current where component amplitudes are comparable with noise levels. The conducted research also showed that the results for the CCCS3 were far below reasonable for any diagnostic system.

From the definitions of bicoherence and tricoherence, it is clear that the analyzed spectral components must exhibit statistical dependencies in order to obtain non-zero results, which are associated with bearing faults. However, bicoherence and tricoherence could be used in specific cases in which fault-related spectral components follow “the bicoherence and the tricoherence frequency rules” [77,78,79]. These higher-order spectra frequency requirements may not be met for all spectral components, which carry valuable information for a fault diagnosis. Therefore, these higher-order spectra frequency requirements limit the usage of spectral components generated by faults, which could be used for a fault diagnosis via motor current signature analysis and thus limited fault types, which could be effectively diagnosed by higher-order spectra.

The proposed cross-correlations do not need to follow “the bicoherence and the tricoherence frequency rules.” Contrary to the bicoherence, tricoherence, and other higher-order spectra, the CCSM technologies allow to take into account statistical dependencies between any needed combinations of multiple spectral components appearing due to a fault, without limitations, i.e., regardless of dependencies between their central frequencies.

Compared to existing technologies, the CCSM technologies also have the advantage that they can be applied by acquiring motor current signals, without the need to install sensors outside the motor electrical box. Indeed, motors are widely used in harsh environments, which makes installation of sensors on a motor very difficult or even impossible. In addition, unlike other diagnostic technologies, the collection of motor stator current data will offer the possibility of diagnosing various possible motor faults, not only bearing faults. This is possible using various MCSA-based technologies. The minor disadvantage of the proposed technologies is a relatively high computational complexity related to the diagnostic feature estimation, which could be easily overcome by powerful IT tools.

Data diversity should be addressed by proper training of the proposed technologies, taking into account multiple faults and possible combinations of the speed and load of various rotating machinery. One needs to obtain proper training data related to these combinations of speed and load in order to reliably diagnose various faults.

If a failure appears due to an unknown fault, the key step in generalizing/investigating the unknown fault is to perform the nine key steps of a classical failure analysis. These steps should be sufficient to define an unknown fault and a failure mode of that fault. In addition, in order to generalize possible known and unknown faults, one needs to create/use a generalized, comprehensive theoretical model of faults.

Finally, the comprehensive validation and comparison of the novel cross-correlation technologies confirmed an important, non-traditional, novel outcome of this research: the technologies based on cross-correlations of spectral moduli were more effective for damage diagnosis than the technologies based on cross-correlations of complex spectra. This non-traditional outcome will allow wide industrial implementation of the novel cross-correlations of spectral moduli for predictive maintenance of various rotating machinery.

## 6. Conclusions

This study comprehensively investigated and validated for the first time by modeling and experiments new, online, nonlinear, higher-order CCSM technologies for the fault diagnosis of rotating machinery. The main contributions of this paper are the novel modeling validation of the technologies, novel experimental validation of the technologies, and novel comparisons with advanced higher-order CCCS technologies.

Experimental trials were undertaken using a test rig dedicated for IM testing. Motor current signals were captured for pristine and damaged bearings. Experimental validation was performed for four pristine tested bearings, two bearings with inner race damage at a 1.2% relative fault severity ratio, and two bearings with outer race damage at relative fault severity ratios of 2.23% and 0.74%.

Experimental and simulation validation confirmed the high probability of correct diagnosis via the CCSM at early-stage fault development. The average total probability of incorrect diagnosis for the CCSM3 technology for all experimental results of 8 tested bearings, estimated via 6528 diagnostic features, was 1.5%. The average total probability of incorrect diagnosis for the CCSM3 technology, estimated via 2400 simulated signals, was 4.3%. The average total probability of incorrect diagnosis for the CCSM4 technology, estimated via 2400 simulated signals, was 5%.

The effectiveness gains in the total probability of incorrect diagnosis for the CCSM3 in comparison with the CCCS3 were 26.8 for the experimental validation and 18.9 for the simulation validation. The effectiveness gains in the Fisher criterion for the CCSM3 in comparison with the CCCS3 were 50.7 for the simulation validation and 104.7 for the experimental validation.

Finally, the comprehensive validation and comparison of the novel cross-correlation technologies confirmed an important non-traditional novel outcome: the technologies based on cross-correlations of spectral moduli were more effective for damage diagnosis than the technologies based on cross-correlations of complex spectra.

The presented higher-order spectral technologies are very powerful technologies for performing damage diagnosis in various electromechanical systems using motor current signature analysis (MCSA). The proposed technologies effectively work even for signals with low signal-to-noise ratio. For the presented research, the main limitations are as follows:

- the quality of data capture equipment. A low noise current transducer (i.e., self-noise was −120 dB in the whole frequency range) and 24-bit DAQ are minimal requirements for current data capture
- the computational complexity of the estimations of the proposed technologies. This limitation is easy to overcome via powerful IT tools.

The proposed technologies are novel, as these technologies do not use a phase spectrum. This non-traditional outcome will open the door for wide industrial implementation of the novel cross-correlations of spectral moduli for predictive maintenance of various rotating machinery. Therefore, the future scope of the research is to prove its effectiveness for other MCSA and vibration applications.

The results of this study are significant for damage diagnosis. The proposed cross-correlations of spectral moduli present a novel conceptualization and will make a considerable impact on damage diagnosis in electrical and mechanical engineering via motor current signature analysis, vibration analysis, ultrasound analysis, etc.

## Figures and Tables

**Table 1:** List of damaged bearings with introduced damage descriptions and fault severity ratios.

Bearing	Introduced Damage	Fault Severity Ratio [19]	Figure
in1	inner race pit damage with diameter of 1 mm and depth of 0.5 mm	1.20%	Figure 8A
in2	inner race scratch damage across the bearing rolling direction with length of 3 mm, width of 1 mm, and depth of 0.7 mm	1.20%	Figure 8B
out1	outer race scratch damage along the bearing rolling direction with length of 3 mm, width of 1 mm, and depth of 0.5 mm	2.23%	Figure 8C
out2	outer race scratch damage across the bearing rolling direction with length of 3 mm, width of 1 mm, and depth of 0.5 mm	0.74%	Figure 8D

**Table 2:** Total number of computational operations for the CCSM3 depending on sampling frequency.

Sampling Frequency [Hz]	Total Number of Operations
65,536	310,778,788
32,768	155,458,468
16,384	77,798,308
8192	38,968,181

**Table 3:** Technology comparison.

	FC			TPCD		
	CCSM3	CCCS3	Gain	CCSM3	CCCS3	Gain
Simulations	5.07	0.1	50.7	4.3%	49%	11.4
Inner race experiments	19.5	0.2	97.5	1.4%	39%	27.9
Outer race experiments	13.2	0.03	440.0	2%	45%	22.5

## Data Availability

Not applicable.

## Nomenclature

CCSM	cross-correlation of spectral moduli
CCSM3	cross-correlation of spectral moduli of order 3
CCSM4	cross-correlation of spectral moduli of order 4
CCCS	cross-correlation of complex spectra
CCCS3	cross-correlation of complex spectra of order 3
CCCS4	cross-correlation of complex spectra of order 4
SNR	signal-to-noise ratio
IM	induction motor
RSH	rotor slot harmonics
PDF	probability density function
TPCD	total probability of correct diagnosis
FC	Fisher criterion

## Appendix A

**Table A1:** List of spectral components used for the inner race diagnosis.

Correlation Number	i	j	k
1	3	−3	−3
	6	−3	−3
	9	−3	−3
2	3	−1	−3
	6	−1	−3
	9	−1	−3
3	3	1	−3
	6	1	−3
	9	1	−3
4	3	3	−3
	6	3	−3
	9	3	−3
5	3	−3	−2
	6	−3	−2
	9	−3	−2
6	3	−1	−2
	6	−1	−2
	9	−1	−2
7	3	1	−2
	6	1	−2
	9	1	−2
8	3	3	−2
	6	3	−2
	9	3	−2
9	3	−3	−1
	6	−3	−1
	9	−3	−1
10	3	−1	−1
	6	−1	−1
	9	−1	−1
11	3	1	−1
	6	1	−1
	9	1	−1
12	3	3	−1
	6	3	−1
	9	3	−1
13	3	−3	0
	6	−3	0
	9	−3	0
14	3	−1	0
	6	−1	0
	9	−1	0
15	3	1	0
	6	1	0
	9	1	0
16	3	3	0
	6	3	0
	9	3	0
17	3	−3	1
	6	−3	1
	9	−3	1
18	3	−1	1
	6	−1	1
	9	−1	1
19	3	1	1
	6	1	1
	9	1	1
20	3	3	1
	6	3	1
	9	3	1
21	3	−3	2
	6	−3	2
	9	−3	2
22	3	−1	2
	6	−1	2
	9	−1	2
23	3	1	2
	6	1	2
	9	1	2
24	3	3	2
	6	3	2
	9	3	2
25	3	−3	3
	6	−3	3
	9	−3	3
26	3	−1	3
	6	−1	3
	9	−1	3
27	3	1	3
	6	1	3
	9	1	3
28	3	3	3
	6	3	3
	9	3	3

**Table A2:** List of spectral components used for the outer race diagnosis.

Correlation Number	i	j
1	4	−5
	4	−3
	4	−1
2	4	1
	4	3
	4	5
3	8	−5
	8	−3
	8	−1
4	8	1
	8	3
	8	5
